# Identification and application of an endophytic fungus *Arcopilus aureus* from *Panax notoginseng* against crop fungal disease

**DOI:** 10.3389/fpls.2024.1305376

**Published:** 2024-02-07

**Authors:** Diangang Sun, Fengyang Li, Lingling Wang, Ruige Chen, Feng Liu, Liwei Guo, Na Li, Fuxian Zhang, Liancheng Lei

**Affiliations:** ^1^ College of Agriculture, Yangtze University, Jingzhou, Hubei, China; ^2^ State Key Laboratory for Zoonotic Diseases, College of Veterinary Medicine, Jilin University, Changchun, Jilin, China; ^3^ College of Animal Science, Yangtze University, Jingzhou, Hubei, China

**Keywords:** endophytic fungus of *Panax notoginseng*, extraction, antifungal, antioxidant, biological control

## Abstract

Endophytic fungi are important microbial resources for developing novel antibacterial and antifungal drugs to prevent and control crop diseases. *Panax notoginseng* has been used as a Chinese medicinal herb for a long time, as it has various bioactivities. However, information on endophytic fungi isolated from *Panax notoginseng* is rare. In this study, an endophytic fungus known as SQGX-6, which was later identified as the golden hair fungus *Arcopilus aureus*, was isolated from *Panax notoginseng*. SQGX-6 was extracted using ethyl acetate, and the active components of the fungus were identified using ultra-performance liquid chromatography-mass spectrometry (UHPLC-MS). The antifungal and antioxidant activities of the extract were determined and evaluated *in vitro* and *in vivo*. SQGX-6 and its extract inhibited the growth of Corn stalk rot (*Fusarium graminearum*), Corn southern leaf blight (*Helminthosporium maydis*), and Tomato gray mold (*Botrytis cinerea*) *in vitro*. The free radical scavenging rates for 2,2-Diphenyl-1-pyridinyl hydrazide (DPPH) radical scavenging activity, 3-Ethylbenzothiazoline-6-Sulfonic Acid Radical scavenging (ABTS) activity were also downregulated by the SQGX-6 extract. *In vivo*, the SQGX-6 extract inhibited the mycelial growth rates of the three aforementioned fungi and downregulated malondialdehyde (MDA) content and upregulated peroxidase (POD) and phenylalanine ammonia-lyase (PAL) content in fruits, leading to significant reduction in damage to cherry tomatoes caused by *Botrytis cinerea*. UHPLC-MS was performed to identify various active substances, including Alkaloids, Azoles, Benzofurans, Coumarins, Flavonoids, Organic acids, Phenols, and plant growth regulators contained in the extract. These results suggested that the endophytic fungus SQGX-6 of *Panax notoginseng* and its extract have excellent antifungal and antioxidant activities, and thus, it is an important microbial resource for the developing novel drugs against plant fungal infections.

## 1 Introduction

Crop diseases caused by bacterial and fungal plant pathogens result in huge economic losses in agriculture every year, which is a major problem that needs to be solved (Rahman et al., 2023). Antibiotics are chemical substances that can prevent or treat diseases caused by bacteria and fungi. However, the overuse of antibiotics has led microbial pathogens to evolve and exhibit resistance to antibiotics, leading to the emergence of drug-resistant “Superbugs” and “Super fungi” around the world. Antibiotic resistance seriously threatens global public health and economic stability (Manganyi and Ateba, 2020). Traditional agricultural chemical bactericides not only contaminate the soil and the environment but also facilitate the selection of antibiotic-resistant bacteria (Nagarajan et al., 2022).

Natural products of microbial origin are an important resource for the development of novel antimicrobial compounds as they have different types of chemical structures and bioactivities (Keller, 2019). Researchers have discovered over 1,500 compounds isolated from fungi between 1993 and 2001, half of which were found to have antibacterial, antifungal, or antitumor activities (Mao et al., 2021). Many researchers in the field of agriculture have screened, identified, and described antimicrobial substances with unique structures and exceptional biological activities from natural products in recent years (Omomowo and Babalola, 2019).

Plant endophytic fungi are microorganisms that live in plant tissues at a certain stage of the life cycle of the plant without causing disease symptoms (Sutton, 1982). Studies have shown that many endophytic fungi stimulate the growth of the host plant, including promoting the growth of medicinal plants, enhancing tolerance to stress, facilitating the accumulation of bioactive metabolites, and substituting for active components of medicinal plants (Aly et al., 2010). Endophytic fungi cultivated from medicinal plants can produce antifungal substances, such as Terpenoids (Liu et al., 2019), Saponins (Yan et al., 2019), Steroids (Hu et al., 2017), Alkaloids (Cui et al., 2017), Peptides (Latz et al., 2018), and Aromatics (Tanney et al., 2016). Therefore, studying endophytic fungi in medicinal plants might help researchers discover new antifungal drugs. *Panax notoginseng*, also known as “Tian Sanqi” or “Shen Sanqi”, is a valuable traditional Chinese medicinal herb. It was first reported in the “Compendium of Materia Medica” and its roots have hemostatic effects (Jiang et al., 2022). They can also promote blood circulation, reduce swelling, and provide pain relief (Sun et al., 2010). *Panax notoginseng* also has antioxidant, anti-diabetic, and antitumor activities (Xie et al., 2017). However, reports on endophytic fungi isolated from *Panax notoginseng* are rare.

Medicinal plants and their associated microflora are important resources for the development of antioxidant products, and antifungal and anticancer drugs (Luo et al., 2023; Yang et al., 2023). However, information on endophytic fungi associated with the medicinal plant *Panax notoginseng* is limited. In this study, we used *Panax notoginseng* cultivated in Yunnan, China, as a material to isolate and identify endophytic fungi with antimicrobial properties in plants. An endophytic fungus, *Arcopilus aureus* SQGX-6, from *Panax notoginseng* was extracted using ethyl acetate, and its antifungal and antioxidant activities against pathogenic fungi were evaluated *in vitro* and *in vivo*. These results can not only enrich the microbial strain resource bank but also provide insights into the use of green micro-ecology to prevent and control plant diseases and contribute to the development of new natural antifungal products.

## 2 Materials and methods

### 2.1 Isolation of endophytes

Endophytes were isolated following the method described in another study (Doin de Moura et al., 2021). *Panax notoginseng* is a perennial herbaceous plant that belongs to the family *Panaxaceae*. We collected five fresh three-year-old *Panax notoginseng* from Yanshan County, Wenshan Zhuang and Miao Autonomous Prefecture, Yunnan Province, China (23.60° N and 104.33° E) and stored them in fresh-keeping bags for 48 h. Then, they were sent to the laboratory for treatment. More information on *Panax notoginseng* is provided in **Supplementary Figure S1**. The roots were rinsed with 75% ethanol for 30 s, and then, rinsed thrice with sterile water. After another rinsing with a 3% sodium hypochlorite solution for 2 min, the roots were washed with sterile water five times. The roots were placed on sterile absorbent filter paper and inoculated with sterile forceps onto a potato dextrose agar (PDA) medium until single fungal colonies were obtained.

### 2.2 Identification of the endophyte SQGX-6

The morphological characteristics of the isolated fungal colonies were analyzed using a light microscope and a scanning electron microscope after the colonies were grown at 28°C for seven days on a PDA medium (Wang et al., 2016). For identification of SQGX-6, specific primers ITS1 (5′-TCCG TAGGTGAACCTGCGG-3′) and ITS4 (5′- TCCTCCGCTTATTGATATGC -3′) were designed, based on the conserved sequences of some known fungi, for PCR (Matar et al., 2022; Nartey et al., 2022). The PCR products were sequenced (Sangon, Shanghai), and the sequencing results were BLAST searched to identify homologs in the NCBI database.The phylogenetic tree was constructed using the N-J method in MEGA-X (Vidal et al., 2020). The sequence was submitted to GenBank (accession number OR053652.1). The fungal strain SQGX-6 was deposited in the China Center for Type Culture Collection (CCTCC) on May 11, 2021, at Wuhan University, Wuhan, Hubei Province, China, with the deposit number of CCTCCM 2022601.

### 2.3 Plate confrontation method

The antifungal activity of SQGX-6 was assessed using the plate confrontation method (Vidal et al., 2020). The source of the pathogenic fungi *F. graminearum*, *H. maydis*, and *B. cinerea* used in this study was donated by Zhang Xiaolei, an associated professor of the College of Agriculture, Yangtze University. An agar plug from a five-day-old fungal pathogen colony was placed on one side of a Petri dish (90 mm diameter), with an agar plug of each SQGX-6 placed on the opposite side, 5 cm from the pathogen. PDA plates inoculated with the pathogen alone were used as the control. The opposing plates were placed in an incubator at 28°C and constant humidity for seven days in the dark. The colony diameter of the pathogen was measured after seven days and compared to the control. The inhibition percentage was calculated according to the following formula:


Inhibition rate (%) = (Rc-Ri) / Rc ×100

Here, Ri indicates the distance between the radial mycelial growth of the pathogenic fungus and the endophytic fungus and Rc indicates the distance between the radial mycelial growth of the pathogenic fungus on the control plate (Chen et al., 2018; Kim et al., 2023). The experiment was repeated three times.

### 2.4 Preparation of endophytic fungal culture medium and ethyl acetate extract

The endophytic fungal culture medium and ethyl acetate extract were prepared based on previous reports. Five pieces of SQGX-6 of a similar size were collected using a drill (6 mm), placed in a PDB liquid medium (50 mL/250 mL) as seed, and cultivated at 28°C for seven days. After cultivation, the seed was inoculated in a 10% ratio in a PDB liquid medium (500 mL/1000 mL) at 28°C for another 7–10 days. The culture medium and mycelium were filtered using a Brinell funnel and extracted thrice with ethyl acetate (3 × 500 mL). The upper phase was collected, decompressed and concentrated using a rotary evaporator. After dissolving with trace methanol, the concentrated solution was vacuum-frozen and dried in a 10 mL ampoule bottle for 72 h, and then, stored in a refrigerator at 4°C (Aqueveque et al., 2017; Zeng et al., 2019).

### 2.5 Determination of the antioxidant activity of the SQGX-6 extract

The SQGX-6 extract was adjusted to five different concentrations (60, 80, 100, 200, and 400 μg/mL) for the antioxidant activity assays: 2,2-Diphenyl-1-pyridinyl hydrazide (DPPH) radical scavenging activity, 3-Ethylbenzothiazoline-6-Sulfonic Acid Radical scavenging (ABTS) activity, and superoxide anion radical (O_2_^−^) scavenging activity; ascorbic acid (VC) was used as a positive control (Li et al., 2022a; Wu et al., 2022). The equations used to evaluate the different scavenging activities are as follows:


DPPH scavenging activity (%) = [(A0−A1)/A0] × 100

Here, A0 indicates the absorbance of the control group, and A1 indicates the absorbance of the extraction solution/standard.


ABTS scavenging activity (%) = [(A-B)/A] × 100

Here, A indicates the absorbance of ABTS and B indicates the absorbance of the combination of ABTS and the test sample.


$$O_{2}^{-}\;\;\mathrm{scavenging}\;\mathrm{activity}\;\left(\%\right)\;=\;\left[\left(A0-A1\right)/A0\right]\;\times \;100$$

Here, A0 indicates the absorbance of pyrogallol, and A1 indicates the absorbance of the sample after 5 min of reaction.

### 2.6 Growth rate inhibition analysis

The antifungal effects of the three pathogenic fungi were evaluated by the growth rate method (Xu et al., 2021; Qi et al., 2022; Zhang et al., 2022). The pathogenic fungal agar plugs (6 mm in diameter) were placed at center of each PDA plate and different concentrations of SQGX-6 extract (8 µg/mL, 16 µg/mL, 32 µg/mL, 64 µg/mL, 128 µg/mL, 256 µg/mL, and 512 µg/mL) were added. The plates were incubated at 28°C in a constant temperature and humidity chamber for seven days, using sterile distilled water as the negative control. The diameter of each colony was measured when the negative control dish was filled with fungi. The inhibition rate of the extract on mycelial growth was calculated using the following equation:


Inhibition rate (%) = (control colony growth distance - treatment colony growth distance) / control colony growth distance × 100

### 2.7 Application of SQGX-6 in plant disease control

The bacteriostatic effect of the SQGX-6 extract was examined *in vivo* as described in another study (Mariana Medina-Romero et al., 2017). Cherry tomatoes (n = 20; diameter: 2–3.5 cm; weight: 7–9 g) with no diseases or wounds in the local area were selected. They exhibited no visible signs of diseases or wounds on their surfaces. The tomato surface were disinfected with 75% ethanol for 2 min, rinsed thrice with sterile distilled water for 60 s each, and then, air-dried on a horizontal flow clean bench. A punch was used to create a hole (4 mm in depth and 6 mm in diameter) in the center of each tomato fruit. Subsequently, 20 µL of SQGX-6 extract at different concentrations (16 µg/mL, 64 µg/mL, and 256 µg/mL) were injected into each wound, with sterile distilled water used as the negative control. After the samples were placed for 1 h in the horizontal flow clean, each wound was inoculated with 20 μL (1 × 10^5^ spores/mL) of gray mycosis spore suspension, forming the control group. Five tomatoes were selected in each treatment group. After air drying at room temperature, the tomatoes were placed in a light incubator, where they were exposed to 12 h of fluorescence and 12 h of dark light, and stored at 25°C for eight days. The diameter of each colony of the gray mold fungal pathogen on tomatoes was measured vertically using a Vernier caliper every two days. Cherry tomato samples were collected after lesions and seeds were removed, and they were flash-frozen using liquid nitrogen. The inhibition rate was calculated using the following equation:

Inhibition rate (%) = (A/B)/A × 100 , where A and B represent the diameter of tomato lesions in the positive control samples and different treatment groups, respectively.

### 2.8 Disease resistance induction assay

After inoculation with 16 µg/mL, 64 µg/mL, and 256 µg/mL of SQGX-6 extract, cherry tomatoes were collected for defense-associated enzyme activity assays. Three enzymes, including malondialdehyde (MDA), peroxidase (POD) and phenylalanine ammonia-lyase (PAL) were tested (Li et al., 2019). Images were taken every two days. About 0.1 g of cherry tomato tissue was taken and 1 mL of the extract was added and homogenized in an ice bath. After grinding, the homogenate was centrifuged at 8,000 *g* at 4°C for 10 min and the supernatant was collected and placed on ice for analysis. The activities of MDA, POD and PAL were measured using MDA, POD, and PAL activity kits (Solebro, Beijing), respectively, following the instructions of the manufacturers. The contents of MDA were expressed in nmol/g mass, and the activities of POD and PAL were expressed in U/g FW.

### 2.9 Identification of the components in antifungal compounds

Ultra-performance liquid chromatography-mass spectrometry (UHPLC-MS) (Sciex Exion LC AD and QTrap 6500+) was performed to analyze the chemical composition of the endophytic fungal SQGX-6 extract. In total, 20 mg of the sample was extracted in a centrifuge tube using 500 μL of a pre-cooled methanol solution (methanol: water = 3:1, v/v, including an internal standard) and incubated at 4°C overnight. After the samples were centrifuged for 15 min at 4°C and 12,000 rpm, the supernatant was collected and filtered through a 0.22 µm filter (Millipore, USA). The flow-through was diluted in 10-fold with a methanol solution and stored at –80°C until further analysis. The liquid chromatography mobile phase conditions are shown in **Supplementary Table S1**. The samples were separated by UHPLC with an EXIONLC System (Sciex). Mobile phase A consisted of 0.1% formic acid in water, and mobile phase B consisted of acetonitrile. The column temperature was maintained at 40°C. The autosampler temperature was maintained at 4°C, and the injection volume was 2 μL. All buffers or solutions used were MS grade.

### 2.10 Statistical analysis

All experiments were conducted in triplicate, and the results were expressed as the mean ± standard deviations (SD). The differences between different groups of data were determined by conducting Duncan’s multiple comparison test using SPSS 17.0 (SPSS Inc., Chicago, USA). All differences between groups were considered to be statistically significant at *P*< 0.05.

## 3 Results

### 3.1 Isolation and screening of antagonistic endophytic fungi

After washing the roots and root tips of *Panax notoginseng*, the water used to rinse the parts for the last time was collected and cultivated on the PDA plates for seven days. No microbial growth was occurred on the surface of the PDA plates, which indicated that the surface of the *Panax notoginseng* roots and root tips were sterilized effectively (data not shown). Subsequently, the roots were placed on a PDA medium and cultivated until fungal colonies were obtained. In total, 24 endophytic fungi were isolated from Yanshan County, Wenshan Zhuang and Miao Autonomous Prefecture, Yunnan Province, China. To screen for the endophytic fungi with antifungal properties, the 24 fungi isolated were co-cultured with pathogenic fungi. We found that one of the fungi showed considerably higher antifungal activity than the others and designated it as SQGX-6 (**Supplementary Figure S2**).

**Figure 1:**
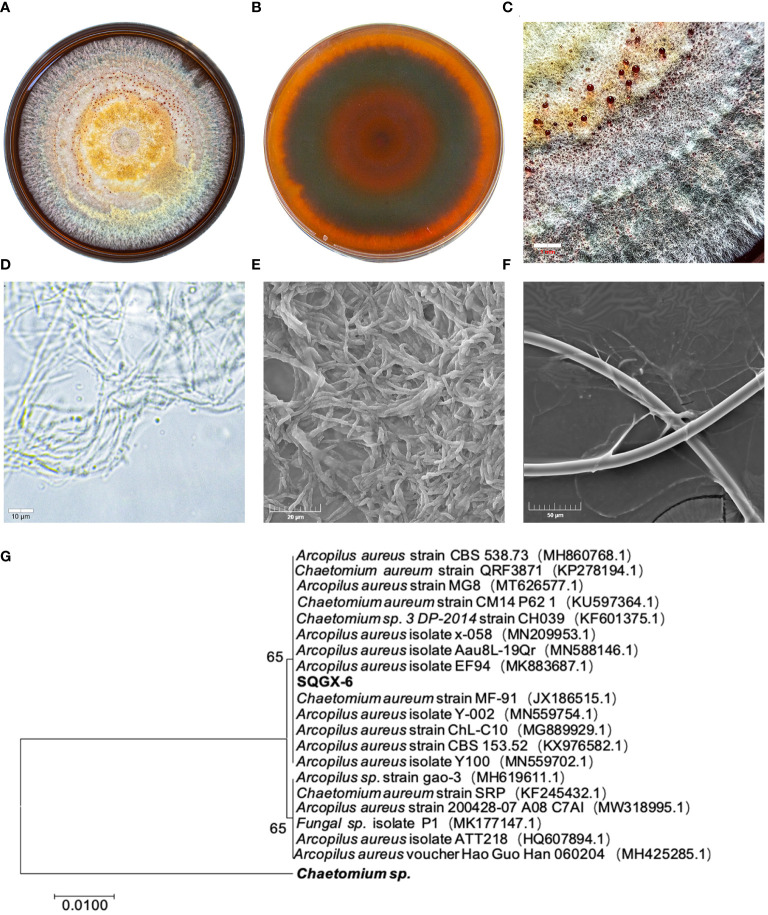
Morphological characteristics of the SQGX-6 strain were assessed using PDA medium and evaluation of growth via scanning electron microscopy, molecular biological identification and phylogenetic tree construction. **(A)** Front side of the SQGX-6 endophytic fungus after 15 days. **(B)** Back side view of the SQGX-6 endophytic fungus at the same age. **(C)** Partial enlarged view of the SQGX-6 Endophytic Fungus (bar = 1 mm). **(D)** Features observation of SQGX-6 mycelia under an optical microscope (bar = 10 μm). **(E, F)** SEM images of SQGX-6, with scale bars of 20 μm in **(E)** and 50 μm in **(F)**. **(G)** Phylogenetic evolution of *Panax notoginseng* endophytic fungus based on the ITS sequence.

We found that the isolate SQGX-6 had typical morphological characteristics of endophytic fungi. After 15 days of culture, the colony showed a cotton-like morphology, ranging in color from orange-yellow to red, with inconspicuous alternating orange-yellow and red concentric rings, which appeared as a wheel (**Figures 1A, B**). Secreted brown-red pigments and mature teardrop-shaped conidia were also observed (**Figure 1C**). Under the light microscope, dense mycelium and spores of SQGX-6 were observed (**Figure 1D**). Scanning electron microscopy (SEM) images of SQGX-6 showed the three-dimensional structure of the mycelial clusters, and the fungal hyphae were prominent. The hyphae had a uniform thickness, were separated regularly like bamboo joints, and were arranged in a rope-like manner (**Figures 1E, F**). We also found straight or curly branches that gradually tapered to the tip.

To confirm the phylogenetic relationship, a phylogenetic tree was constructed using the MEGA-X software, and SQGX-6 was compared to its closely related strains. The homology with *Arcopilus aureus* was 100% (**Figure 1G**). Thus, this endophytic fungus belonged to the phylum *Ascomycetes*, class *Sordariomycetes*, order *Sordariales*, family *Chaetomiaceae*, and genus *Arcopilus.*

### 3.2 SQGX-6 inhibited the growth of pathogenic fungi

**Figure 2:**
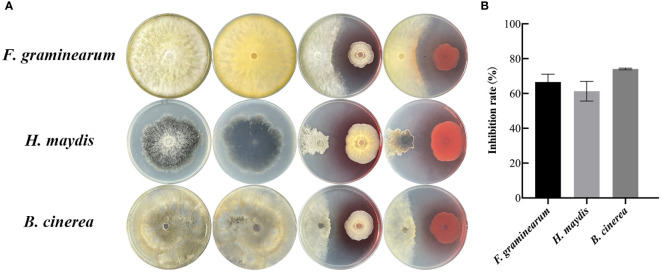
Confrontation experiment between the endophytic fungus SQGX-6 and three other pathogenic fungi. **(A)** Corn stalk rot (*Fusarium graminearum*), corn southern leaf blight (*Helminthosporium maydis*), and tomato gray mold (*Botrytis cinerea*). **(B)** Antifungal activity of SQGX-6 against these three pathogenic fungi.

To determine whether the endophytic fungus SQGX-6 has the antifungal effects, three common crop pathogenic fungi, including Corn stalk rot (*Fusarium graminearum, F. graminearum*), corn southern leaf blight (*Helminthosporium maydis, H. maydis*), and Tomato gray mold (*Botrytis cinerea, B. cinerea*), were co-cultured with SQGX-6 using the plate confrontation method. We found that SQGX-6 inhibited the growth of *F. graminearum*, *H. maydis* and *B. cinerea*, resulting in the atrophy of the pathogenic fungal mycelium after seven days of cultivation on PDA plates (**Figure 2A**). Compared to the inhibition rate in the control group, the inhibition rate of SQGX-6 against the growth of the three pathogenic fungi exceeded 50%, the inhibition rate against *B. cinerea* was the highest (74.07%), and the inhibition rates against the other two pathogenic fungi were 66.61% and 61.30%, respectively (**Figure 2B**). These results suggested that SQGX-6 can strongly inhibit fungi and is a promising antifungal agent.

### 3.3 SQGX-6 extract showed antioxidant activity

**Figure 3:**
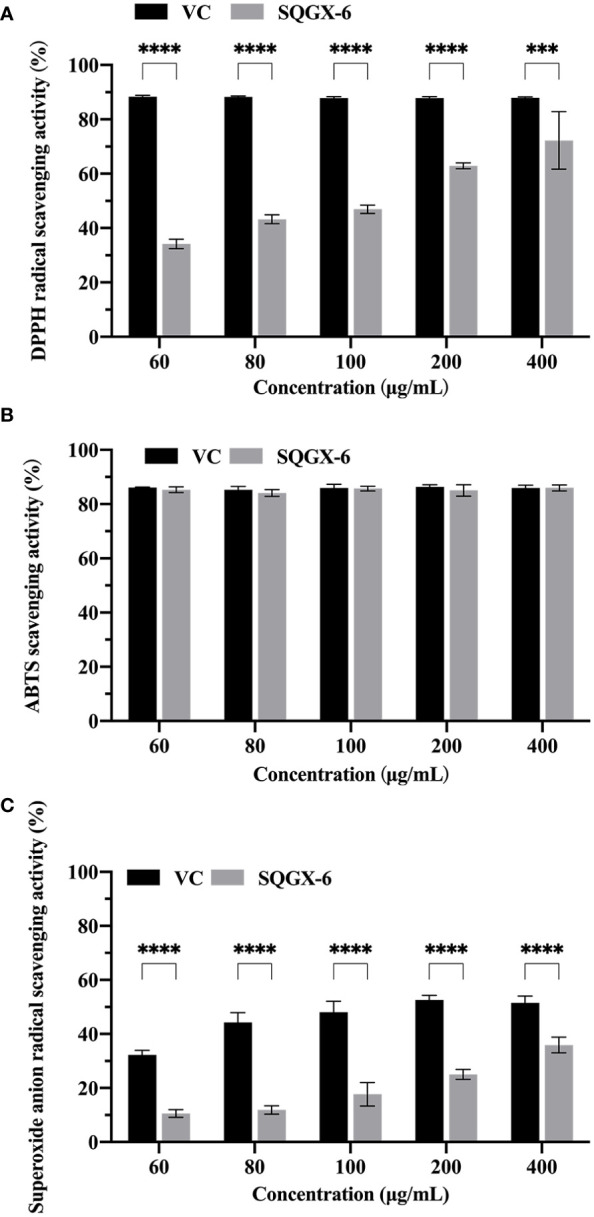
Antioxidant activity of the SQGX-6 extract. **(A)** DPPH radical scavenging activity. **(B)** ABTS radical scavenging activity. **(C)** Superoxide anion radical scavenging activity. Notably, a significant difference was observed between the groups (*P*< 0.001); ***: *P*<0.001; ****: *P*<0.0001.

To evaluate whether the SQGX-6 extract has antioxidant activity, the DPPH radical scavenging test, ABTS free radical scavenging test, and superoxide anion radical scavenging test were performed. DPPH is commonly used to evaluate the free radical scavenging effects of various antioxidants. The results showed that the SQGX-6 extract exhibited strong antioxidant activity in a dose-dependent manner compared to the control, where the highest DPPH radical scavenging rate was 72.23% at 400 μg/mL (**Figure 3A**). The SQGX-6 extract also showed a strong ABTS scavenging activity. The ABTS scavenging activity was above 80% when the concentration of the SQGX-6 extract was 60–400 μg/mL. This activity was the same as that recorded in the VC control group (**Figure 3B**). The scavenging activity on the superoxide anion radicals increased along with the concentration of the SQGX-6 extract. The highest scavenging rate of superoxide anion radicals was 35.90% when the concentration of the SQGX-6 extract was 400 μg/mL (**Figure 3C**). These results showed that the SQGX-6 extract had a high antioxidant activity.

### 3.4 SQGX-6 extract had antifungal effects on pathogenic fungi

**Figure 4:**
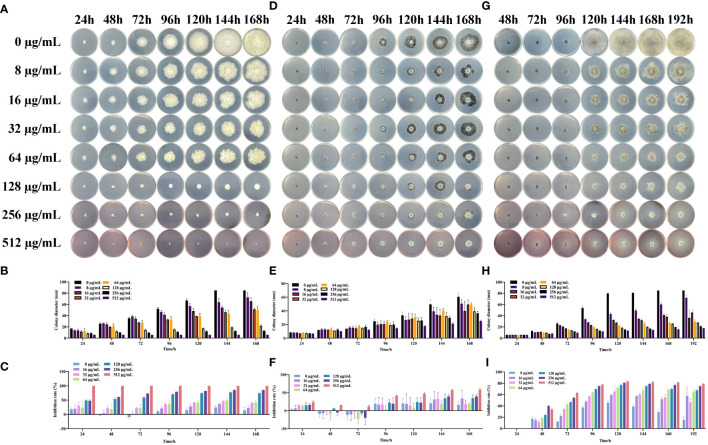
Effects of the SQGX-6 extract on the mycelial growth of three pathogenic fungi. Colony diameters were measured daily over a 7-day period, and the graph’s indicated concentration is the final concentration after the extract was added to the PDA plate. Displayed data represent images from triplicate replicates. **(A–C)** represent *F*. *graminearum*; **(D–F)** to *H*. *maydis*; **(G–I)** to *B*. *cinerea*.

To analyze the effect of the SQGX-6 extract on the growth of crop pathogenic fungi, including *F. graminearum*, *H. maydis* and *B. cinerea*, the pathogenic fungi were cultured on PDA plates with different concentrations of the SQGX-6 extract, and the growth rate was determined by measuring the colony diameters. The SQGX-6 extract inhibited the growth of the three pathogenic fungi (**Figure 4**), which matched the results of the SQGX-6 confrontation experiment. As the concentration and action time of the extract increased, its inhibitory effect on the growth of *F. graminearum* increased. At a concentration of 256 μg/mL, the extract completely inhibited the growth of *F. graminearum*. At 24 h, the inhibition rate of the extract was 66.3% (**Figures 4A, B**), which increased to 90.13% at 168 h (**Figure 4C**). The diameter and growth inhibition rate of *H. maydis* were positively correlated with the concentration and action time of the SQGX-6 extract. At 24 h, the diameter of on *H. maydis* after treatment with 512 μg/mL of the extract was 6.47 mm, and the inhibition rate was 23.62%. At 144 h and 168h, 512 μg/mL of the extract had similar inhibition rates of 57.19% and 58.67%, respectively for *H. maydis* (**Figures 4D, F**). Similar results were observed for the fungus *B. cinerea*. At 72 h, the inhibition rate of the 512 μg/mL of the extract against *B. cinerea* was 63%. At 120 h, the inhibition rate was the highest at 83.30%. However, the inhibition rate decreased slightly after 120 h, with rates of 82.02%, 81.49%, and 79.08% recorded at 144 h, 168 h, and 192 h, respectively (**Figures 4G–I**). These results indicated that the SQGX-6 extract can inhibit the growth of pathogenic fungi.

### 3.5 SQGX-6 extract inhibited the rotting of artificially damaged cherry tomatoes

**Figure 5:**
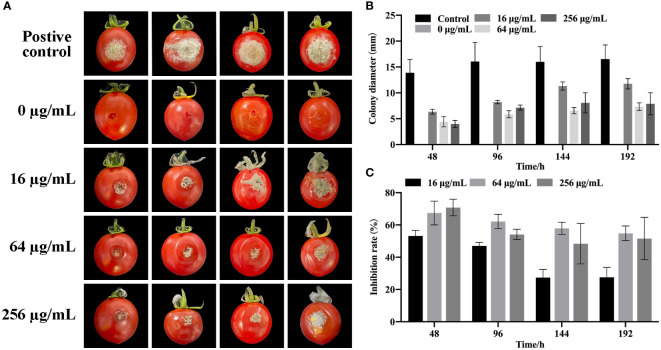
Impact of SQGX-6 treatment on *B*. *cinerea* infection in postharvest cherry tomatoes. **(A)** Inoculation of cherry tomato fruits with a 20 μL *B*. *cinerea* spore suspension at a concentration of 1×10^5^ spores/mL, followed by SQGX-6 treatment at various concentrations (0 μg/mL, 16 μg/mL, 64 μg/mL, or 256 μg/mL) and storage at 25°C. Lesion diameters and disease incidence were recorded on the 8th day post-inoculation. The vertical bar signifies the standard deviation of the mean (n = 5). **(B)** Diameter of SQGX-6 extracts’ inhibitory effect on postharvest *B*. *cinerea* growth in cherry tomatoes. **(C)** Inhibition rate of SQGX-6 extract treatment on postharvest *B*. *cinerea* infection of cherry tomatoes.

After cultivation for eight days at 25°C, no fungi were observed in cherry tomatoes (0 μg/mL) without *B. cinerea*, whereas many fungi were observed in cherry tomatoes inoculated with *B. cinerea* alone (**Figure 5A**). Although large areas of fungi were found in the *B. cinerea* infection group, the growth of *B. cinerea* was substantially inhibited by the SQGX-6 extract in a dose dependent manner. Among which, the most prominent inhibitory effect was observed at 64 μg/mL, the inhibition rate was 67.42%, and the diameter of the fungus was only 4.42 mm (**Figure 5B**). At 48 h, the lesion area of tomatoes in the *B. cinerea* infection group was 13.89 mm in diameter. A higher concentration of the extract significantly alleviated the degree of rot caused by *B. cinerea* compared to the effect of a lower concentration of the extract on the degree of rot (**Figure 5C**). These results showed that treatment with the SQGX-6 extract can inhibit postharvest *B. cinerea* infection in cherry tomatoes.

### 3.6 SQGX-6 extract increased the resistance of cherry tomatoes infected with *B. cinerea*

**Figure 6:**
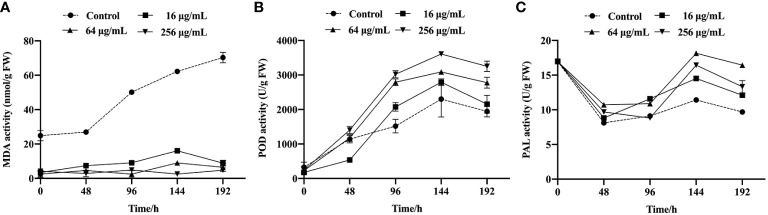
Cherry tomatoes were infected with *B. cinerea* and incubated at 25 °C to study the activities of MDA **(A)**, POD **(B)**, and PAL **(C)** in the fruits, the experiment was conducted for 192 h.

To elucidate the mechanism underlying the antifungal effects of the SQGX-6 extract, the effects of the SQGX-6 extract on the contents of MDA, POD and PAL in cherry tomatoes were further investigated. The results showed that the MDA content of infected cherry tomatoes changed significantly after treatment with 16 μg/mL, 64 μg/mL and 256 μg/mL of the extract, compared to the MDA content in the control group. The MDA content in the control group continued to increase after tomato inoculation, but it did not increase in the groups that received different concentrations of the SQGX-6 extract. Thus, the accumulation of MDA in the SQGX-6 extract treatment group was significantly slower than that in the control group (**Figure 6A**). At 192 h post-infection, the MDA content in the group treated with 256 μg/mL of the extract was approximately 16-fold lower than that in the control group. These results showed that treatment with the SQGX-6 extract significantly decreased the MDA content in cherry tomatoes after harvest.

The POD activity in the control group and the SQGX-6 extract treatment groups increased throughout the infection period (**Figure 6B**). After 96 h of infection, the POD content in the groups treated with 16 μg/mL and 64 μg/mL of the extract was approximately two times higher than that in the control group. From 144 h to the 192 h, the POD activity of each treatment group was significantly higher than that of the control group. These results indicated that the SQGX-6 extract increased the POD activity in cherry tomatoes infected with *B. cinerea*.

Similarly, the PAL content in cherry tomatoes infected with *B. cinerea* increased after treatment with the SQGX-6 extract (**Figure 6C**). At 144 h, when the PAL activity in the group treated with 256 μg/mL of the extract was approximately 1.6 times higher than that in the control group. Over the next two days, the PAL activity decreased in all groups. At 192 h, the PAL activities in the groups treated with 16 μg/mL, 64 μg/mL and 256 μg/mL of the extract were 1.25 times, 1.7 times, and 1.4 times higher than the PAL activity of the control group, respectively (**Figure 6C**). These results suggested that the SQGX-6 extract can enhance the activity of disease-resistant enzymes to induce disease resistance in cherry tomatoes.

### 3.7 UPLC-MS detection of the components in the SQGX-6 extract

**Figure 7:**
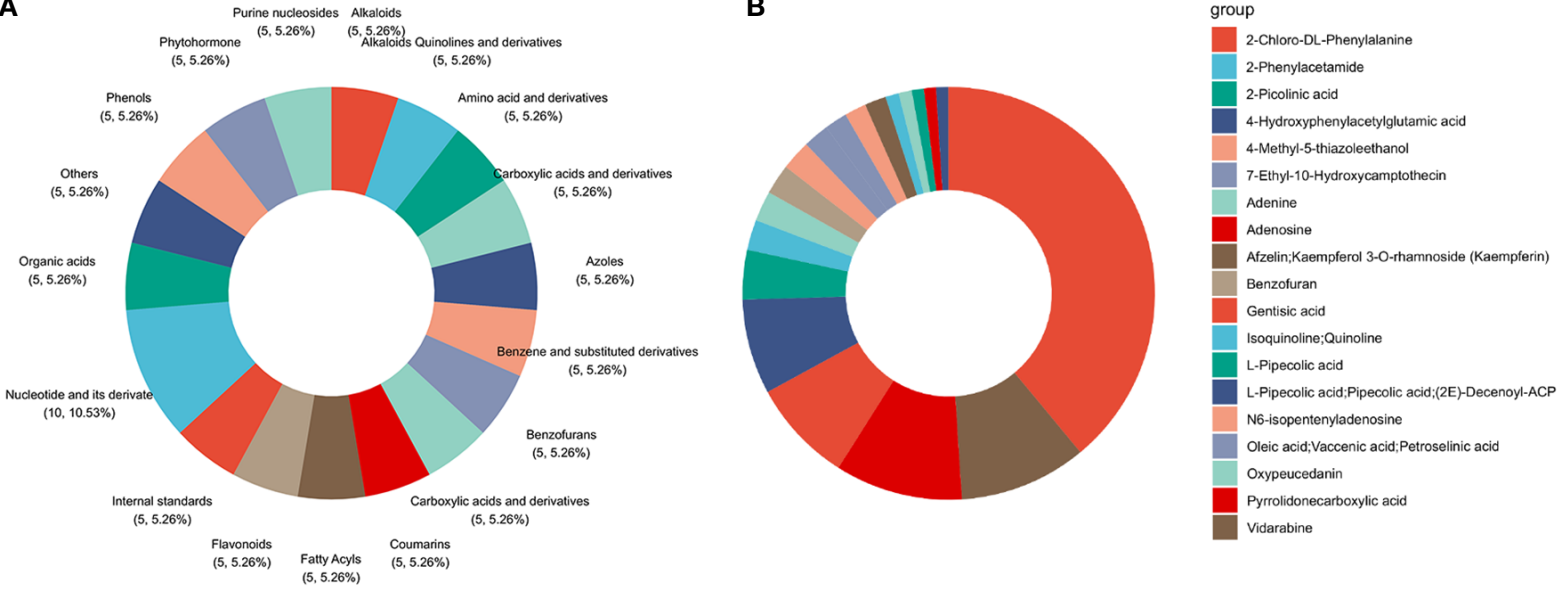
Determination of chemical species and components in the endophytic fungus extract via UHPLC−MS analysis. **(A)** Classification chart of the top twenty extracts. **(B)** These extracts ranked in terms of ingredient contents.

To identify the components of the SQGX-6 extract, it was analyzed using the ultra-performance liquid chromatography-mass spectrometry (UPLC/MS) technique. In total, 618 compounds were identified from the extract (**Supplementary Table S1**). The top 20 compounds, ranked by classification, were Alkaloids, Quinolines and their derivatives, Amino acids and their derivatives, Carboxylic acids and derivatives, Azoles, Benzene and substituted derivatives, Benzofurans, Carboxylic acids and their derivatives, Coumarins, Fatty acids, Acyls, Flavonoids, Nucleotides and their derivatives, Organic acids, Phenols, Phytohormone, Purine nucleosides, and Steroids and their steroid derivatives (**Figure 7A**). According to the ranking based on composition, the top 20 compounds were 2-Chloro-DL-Phenylalanine, Vidarabine, Adenosine, Gentisic acid, 4-Hydroxyphenylacetylglutamic acid, 2-Picolinic acid, 2-Phenylacetamide, Adenine, Benzofuran, N6-isopentenyladenosine, Oleic acid, Vaccenic acid, Petroselinic acid, 7-Ethyl-10-Hydroxycamptothecin, 4-Methyl-5-thiazoleethanol, Afzelin, Kaempferol 3-Orhamnoside (Kaempferin), Isoquinoline, Quinoline, Oxypeucedanin, L-pipecolic acid, Pyrrolidonecarboxylic acid, L-Pipecolic acid, Pipecolic acid, (2E)-Decenovl-ACP, and Nandrolone (**Figure 7B**). The names of the identified chemical components in the extract, along with their KEGG IDs, CAS numbers, molecular weights, and molecular formulae are presented in **Supplementary Table S2**. To summarize, 2-Chloro-DL-Phenylalanine, Vidarabine, and Adenosine were the compounds with the highest concentrations in the extract of the endophytic fungus. This might be closely related to the bioactivity of secondary metabolites produced by the endophytic fungi.

## 4 Discussion

Synthetic pesticides are commonly used after cash crops are cultivated and harvested for treating diseases caused by fungi. However, when using agricultural chemicals, several factors, including the duration and frequency of treatment, concentration, and active ingredients, are often ignored, leading to high production costs (Lydia Macias-Rubalcava et al., 2018). Additionally, developing antifungal crops requires extensive research, application, and production processes, which are time-consuming and expensive (Lydia Macias-Rubalcava et al., 2018). Compared to synthetic pesticides, endophytic fungi are a novel and important microbial resource for producing biologically active compounds. They possess several secondary metabolites and have the advantages of renewable resources and reliable productivity. However, only a few endophytic fungi have been identified. In a study (Jiao et al., 2020), the morbidity of cherry tomatoes decreased to 52.9% after treatment with the endophytic fungus ϵ-PL at a concentration of 3,200 mg/L, compared to the morbidity of cherry tomatoes in the control group. The endophytic fungus H4 has showed high antifungal activity against *Clostridium fragilis* and pretreatment with the H4 extract significantly decreased the severity and morbidity of *Anthrax* (Li et al., 2021). In this study, we identified the novel endophytic fungus SQGX-6. Treatment with SQGX-6 and its extract considerably decreased the time and degree of decay of tomatoes after infection with *B. cinerea*.

SQGX-6 and its ethyl acetate extract inhibited the cell growth of all three pathogenic fungi. The antifungal effect of the extract was better than that of the endophytic fungi, which might be due to the production of a large number of active substances after fermentation (Fatima et al., 2022). By performing UHLC-MS, we found that the extract mainly contained Alkaloids, Azoles, Benzofuran, Coumarin, Flavonoids, Organic acids, Phenols and phytoauxin, which possess various biological activities, including antibacterial, antifungal, immunosuppressive, antiviral, antioxidant, anti-inflammatory and anticancer (Toghueo, 2020). For example, the organic acids isolated from the endophytic mucor of the medicinal plant *Pulsatilla chinensis* showed a weak antifungal effect on *Aspergillus terreus*, with MIC values of 127.8 µM and 111.2 µM, respectively (Abdou et al., 2020). Rosemary extract contains phenolic and flavonoid substances, the extract of which has antifungal effects on *Candida albicans* and *Candida donovani* (Meccatti et al., 2021). This study found that the secondary metabolites of endophytic fungus SQGX-6 extract are very abundant, many of these metabolites which are more effective and safer than traditional antifungal agents (Manganyi and Ateba, 2020).

Different free radicals produce different degrees of oxidative damage, and since a single antioxidant system cannot reflect the biological significance, different complementary systems are needed to determine the real effect of the sample on antioxidants. In this study, the antioxidant activity of the samples was evaluated using three antioxidant indices, including DPPH, ABTS, and superoxide anion radical scavenging capacity. Our results showed that the SQGX-6 extract exhibited high antioxidant activity at specific concentrations, which was comparable to VC. However, further studies are needed to determine the antioxidant potentials of the SQGX-6 extract.

Besides enhancing antifungal effects, inducing resistance is an effective way to control postharvest rot in tomato fruits (Zhu and Zhang, 2016; Debode et al., 2018). Some researchers reported that resistance and defense to pathogenic fungi might be associated with enzyme activities (Prasad et al., 2022). We found that the SQGX-6 extract can induce the resistance of cherry tomatoes to gray mold. The MDA content of cherry tomatoes inoculated with the *Botrytis* tomato fungus after induction by the SQGX-6 extract was significantly lower than that in the control group, indicating that the SQGX-6 extract could inhibited MDA production. A reduction in MDA levels decreased peroxidation damage to the cell membrane, which in turn increased the stress resistance of plants to pathogenic fungi (Song et al., 2014). The contents of POD and PAL in tomato fruits increased after treatment with the SQGX-6 extract. Changes in the PAL activity were strongly correlated with plant resistance and the total phenol content (Bill et al., 2017). Compared to the activity of PAL in the infection group, the activity of PAL was upregulated in the SQGX-6 extract-treated group, which indicated that the SQGX-6 extract stimulated the active defense response of cherry tomatoes and increased the defense of cherry tomatoes against tomato fungi during storage. Therefore, a higher level of defense enzymes might be a key factor in reducing the susceptibility of cherry tomatoes to *B. cinerea* (Li et al., 2022b). A study showed that benzothiadiazole (BTH) functions as an analog of salicylic acid (SA) and can be used to postpone the postharvest decay of various fruits. A study showed that BTH treatment increased the activities of CAT, SOD, PPO, and PAL in lychee fruits, thus increasing their resistance against frost disease after harvest (Xu et al., 2021a).

Microecological control of plant diseases is an eco-friendly approach (Samaras et al., 2021). Due to its broad ecological adaptability, fungi are highly favorable broad-spectrum agents for the control of plant diseases. These products are mainly used in farmland to increase crop yield and protect crops from diseases. In this study, the endophytic fungus SQGX-6 and its extract effectively inhibited three pathogenic fungi that affect crops and also decreased the rotting condition of tomato fruits caused by the tomato gray mold pathogenic fungus. Treatment with different concentrations of the extract can not only improve the POD activity of tomato fruits to a certain extent, thus improving the antioxidant capacity of tomatoes, but it can also delay the rotting process of tomatoes by inhibiting the activities of MDA and PAL activity.

## 5 Conclusion

In this study, an endophytic fungus with antifungal activity was isolated from the Chinese medicinal herb *Panax notoginseng* and was identified as *Arcopilus aureus*. *Arcopilus aureus* and its ethyl acetate extract showed different degrees of bioactivity, including antioxidant activity and inhibitory effects on the growth of *F. graminearum*, *H. maydis*, and *B. cinerea*, which can effectively decrease the postharvest decay of cherry tomatoes caused by pathogenic fungi *in vitro* and *in vivo*. Therefore, organic compounds produced by endophytic fungi might play an important role in the biological control of crop pathogens and might be used as eco-friendly antifungal agents for controlling plant diseases. However, further investigation is needed to develop these antifungal agents.

## Data availability statement

The datasets presented in this study can be found in online repositories. The names of the repository/repositories and accession number(s) can be found below: https://www.ncbi.nlm.nih.gov/genbank/, OR053652.

## Author contributions

DS: Data curation, Formal analysis, Investigation, Methodology, Writing – original draft. FL: Project administration, Supervision, Writing – review & editing. LW: Methodology, Writing – original draft. RC: Investigation, Writing – original draft. FL: Supervision, Writing – original draft. LG: Supervision, Writing – original draft. NL: Supervision, Writing – original draft. FZ: Supervision, Visualization, Writing – review & editing. LL: Conceptualization, Funding acquisition, Resources, Writing – review & editing.

## Supplementary material

Supplementary Figure 1 Three-year-old *Panax notoginseng* from Yanshan County, Wenshan Zhuang and Miao Autonomous Prefecture, Yunnan Province, China (23.60° N and 104.33° E). S1A. Five fresh three-year-old *Panax notoginseng*, the average height is 68-75 cm. S1B. Single *Panax notoginseng*, the number of leaves per plant is about 20-25 at the time of collection. S1C. Close up of the root of *Panax notoginseng*, with a diameter of approximately 15 cm.

Supplementary Figure 2 PCR amplification and agarose gel electrophoresis of endophytic fungus SQGX-6 from *Panax notoginseng*.

Supplementary Table 1 Table of mobile phase conditions for liquid chromatography.

Supplementary Table 2 Identified chemical constituents of bioactive fungal extract.
