# Supplementary material for: Identification and application of an endophytic fungus Arcopilus aureus from Panax notoginseng against crop fungal disease
Source: Front Plant Sci. 2024 Feb 7;15:1305376. doi: 10.3389/fpls.2024.1305376 (PMC10880449; doi:10.3389/fpls.2024.1305376)
Supplement: Supplementary Figure 1 — Three-year-old Panax notoginseng from Yanshan County, Wenshan Zhuang and Miao Autonomous Prefecture, Yunnan Province, China (23.60° N and 104.33° E). S1A. Five fresh three-year-old Panax notoginseng, the average height is 68-75 cm. S1B. Single Panax notoginseng, the number of leaves per plant is about 20-25 at the time of collection. S1C. Close up of the root of Panax notoginseng, with a diameter of approximately 15 cm. [file DataSheet_1.pdf]

Supplementary Material

1 Supplementary Figures and Tables

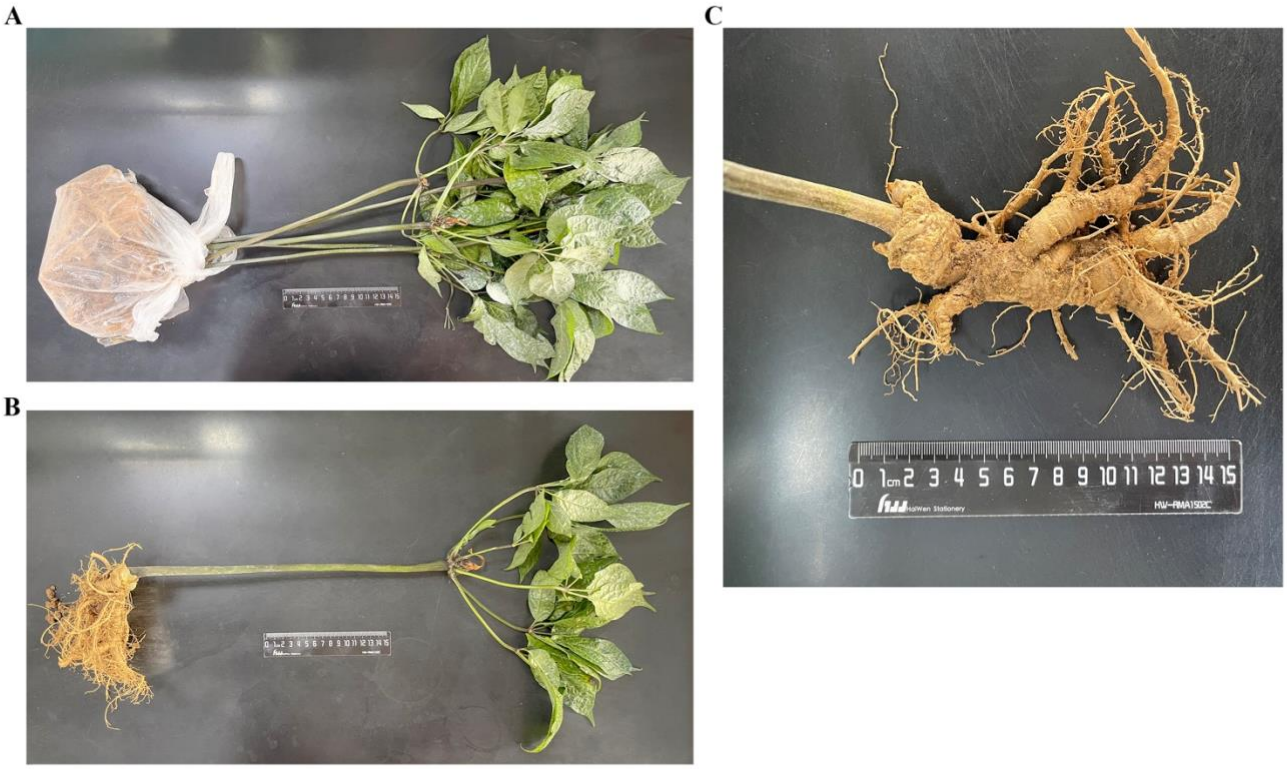

Figure S1. Three-year-old *Panax notoginseng* plants from Yanshan County, Wenshan Zhuang and Miao Autonomous Prefecture, Yunnan Province, China (23.60° N and 104.33° E) were utilized. S1A. Five fresh three-year-old *Panax notoginseng* plants, with an average height of 68-75 cm. S1B. Single *Panax notoginseng*, the number of leaves per plant is about 20-25 at the time of collection. S1C. Close up of the *Panax notoginseng* root, approximately 15 cm in diameter.

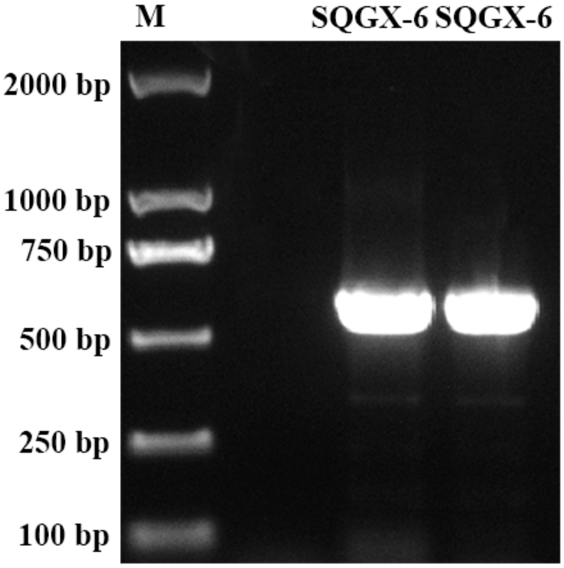

Figure S2. PCR amplification and agarose gel electrophoresis of the endophytic fungus SQGX-6 from *Panax notoginseng*.

Table S1. Mobile phase conditions for liquid chromatography.

Time	Flow rate	A % Water	B % Acetonitrile
0	400	98	2
0.5	400	98	2
10	400	50	50
11	400	5	95
13	400	5	95
13.1	400	98	2
15	400	98	2

Table S2. Chemical constituents of bioactive fungal extracts.

	NAME	CAS	KEGG_ID	FORMULA	EXACT_MASS	CLASS	Retention Time	Ionmode
1	Chrysin	480-40-0	C10028	C15H10O4	254.0579	flavonoids	10.94	+
2	Indole-3-carboxaldehyde	487-89-8	C08493	C9H7NO	145.0528	phytohormone	6.73	+
3	2-Chloro-DL-Phenylalanine	14091-11-3		C9H10ClNO2	199.040006	internal standards	3.85	+
4	Hyperoside	482-36-0	C10073	C21H20O12	464.0955	flavonoids	6.99	+
5	Isoquercitrin	482-35-9	C05623	C21H20O12	464.0955	flavonoids	6.06	+
6	Indole-3-acetic acid	87-51-4	C00954	C10H9NO2	175.0633	phytohormone	7.14	+
7	Farrerol	24211-30-1	C09734	C17H16O5	300.0998	flavonoids	10.61	+
8	L-Tyrosine	60-18-4	C00082	C9H11NO3	181.0739	amino acids	1.93	+
9	Acacetin	480-44-4	C01470	C16H12O5	284.0685	flavonoids	11.03	+
10	N6-(delta 2-Isopentenyl)-adenine	2365-40-4	C04083	C10H13N5	203.1171	phytohormone	5.3	+
11	cis-Zeatin	32771-64-5	C15545	C10H13N5O	219.112	phytohormone	3.6	+
12	N6-isopentenyladenosine	7724-76-7		C15H21N5O4	335.159354	phytohormone	6.1	+
13	Pinocembrin	480-39-7	C09827	C15H12O4	256.0736	flavonoids	11.09	+
14	Luteolin	491-70-3	C01514	C15H10O6	286.0477	flavonoids	8.03	+
15	Cianidanol	18829-70-4	C06562	C15H14O6	290.079	flavonoids	4.5	+
16	(-)-Epigallocatechin	970-74-1	C12136	C15H14O7	306.074	flavonoids	4.12	+
17	Gallocatechin	970-73-0	C12127	C15H14O7	306.074	flavonoids	3.36	+
18	sinensetin	2306-27-6	C10186	C20H20O7	372.1209	flavonoids	10.34	+
19	Astragalin	480-10-4	C12249	C21H20O11	448.1006	flavonoids	6.52	+
20	Apiin	26544-34-3	C04858	C26H28O14	564.1479	flavonoids	6.46	+
21	Rhoifolin	17306-46-6	C12627	C27H30O14	578.1636	flavonoids	6.5	+
22	Kaempferol-3-O-rutinoside	17650-84-9	C21833	C27H30O15	594.1585	flavonoids	6.29	+
23	Diosmin	520-27-4	C10039	C28H32O15	608.1741	flavonoids	6.59	+
24	Rutin	153-18-4	C05625	C27H30O16	610.1534	flavonoids	5.85	+
25	Icariin	489-32-7	C17555	C33H40O15	676.2367	flavonoids	8.12	+
26	Methyl (indol-3-yl)acetate;Methyl 3-indolylacetate	1912-33-0	C20635	C11H11NO2	189.078979	Alkaloids;phytohormone	9.48	+
27	6-(Furfurylamino)purine;Kinetin	525-79-1	C08272	C10H9N5O	215.08071	Alkaloids;phytohormone	4.55	+
28	Baicalein	491-67-8	C10023	C15H10O5	270.0528	flavonoids	9.38	-
29	Dihydrojasmonic Acid	3572-64-3		C12H20O3	212.141244	phytohormone	9.82	-
30	(+)-Abscisic acid	21293-29-8	C06082	C15H20O4	264.1362	phytohormone	7.86	-
31	Gibberellin A4	468-44-0	C11864	C19H24O5	332.1624	phytohormone	9.98	-
32	Avicularin	572-30-5		C20H18O11	434.084911	flavonoids	6.47	-
33	Liquiritin	551-15-5	C16989	C21H22O9	418.1264	flavonoids	6	-
34	(±)-Jasmonic acid	77026-92-7	C08491	C12H18O3	210.1256	phytohormone	8.94	-
35	N-((-)-jasmonoyl)-S-isoleucine	120330-92-9	C18699	C18H29NO4	323.209658	phytohormone	10.41	-
36	Salicylic acid	69-72-7	C00805	C7H6O3	138.031695	Phenols;phytohormone	7.06	-
37	Trimethoprim	738-70-5	C01965	C14H18N4O3	290.137891	Phenol ethers	5.08	+
38	Epitulipinolide	24164-13-4	C09566	C17H22O4	290.15181	Sesquiterpenoids	5.07	+
39	Oxypeucedanin	737-52-0;26091-73-6	C09282	C16H14O5	286.084125	Coumarins	8.12	+
40	4-Methoxyphenyl beta-D-glucopyranoside	6032-32-2	C17599	C13H18O7	286.105255	Phenols	5.52	+
41	2-O-Methylanigorufone	56252-05-2		C20H14O2	286.09938	Phenols	7.57	+
42	Isosakuranetin	480-43-3	C05334	C16H14O5	286.084125	Flavonoids	6.53	+
43	Brazilin	474-07-7	C09920	C16H14O5	286.084125	Flavonoids	6.2	+
44	2',3,5,7-Tetrahydroxyflavone	480-15-9	C10036	C15H10O6	286.04774	Flavonoids	6.53	+
45	Orobol (5,7,3',4'-tetrahydroxyisoflavone)	480-23-9	C10510	C15H10O6	286.04774	Flavonoids	7.9	+
46	Demethoxycapillarisin	61854-36-2	C17786	C15H10O6	286.04774	Flavonoids	6.51	+
47	Citreorosein	481-73-2	C17810	C15H10O6	286.04774	Anthraquinones	7.59	+
48	2'-Hydroxygenistein	1156-78-1	C12134	C15H10O6	286.04774	Flavonoids	7.59	+
49	(-)-Sativan	41743-86-6	C10526	C17H18O4	286.12051	Flavonoids	7.89	+
50	3-Methylxanthine	1076-22-8	C16357	C6H6N4O2	166.049076	Nucleotide and its derivates	2.71	+
51	4-Hydroxyphenyl-2-propionic acid		C03080	C9H10O3	166.062995	Phenylpropanoic acids	10.09	+
52	Acetovanillone	498-02-2	C11380	C9H10O3	166.062995	Phenols	5.24	+
53	Pyrrole-2-carboxylic acid	634-97-9	C05942	C5H5NO2	111.032029	Amino acid and derivatives	2.29	+
54	5-Aminovaleric acid	660-88-8	C00431	C5H11NO2	117.078979	Amino acid and derivatives	1.11	+
55	Guanidineacetic acid	352-97-6	C00581	C3H7N3O2	117.053827	Amino acid and derivatives	1.11	+
56	Toluene-cis-dihydrodiol		C04592	C7H10O2	126.06808		1.78	+
57	D-alpha-Aminobutyric acid	2623-91-8	C02261	C4H9NO2	103.063329	Carboxylic acids and derivatives	0.65	+
58	Urocanic acid	104-98-3	C00785	C6H6N2O2	138.042928	Azoles	4.21	+
59	3-Hydroxybenzoic acid	99-06-9	C00587	C7H6O3	138.031695	Phenols	8.39	+
60	2,5-Dihydroxybenzaldehyde	1194-98-5	C05585	C7H6O3	138.031695	Phenols	5.09	+
61	Styrene-cis-2,3-dihydrodiol		C07084	C8H10O2	138.06808		5.12	+
62	Sesamol	533-31-3	C10832	C7H6O3	138.031695	Phenols	3.34	+
63	Vidarabine	5536-17-4;24356-66-9	C07195	C10H13N5O4	267.096755	Purine nucleosides	2.28	+
64	Adenosine	58-61-7	C00212	C10H13N5O4	267.096755	Nucleotide and its derivates	2.58	+
65	Uridine	58-96-8	C00299	C9H12N2O6	244.069538	Nucleotide and its derivates	2.47	+
66	Panaxynol	81203-57-8	C17447	C17H24O	244.182715	Miscellaneous	12.57	+
67	Pseudouridine	1445-07-4	C02067	C9H12N2O6	244.069538	Nucleotide and its derivates	8.73	+
68	Rhombifoline	529-78-2	C10781	C15H20N2O	244.157563	Alkaloids	8.74	+
69	Batatasin III	56684-87-8		C15H16O3	244.109945	Phenols	8.73	+
70	Cinnamic acid	140-10-3	C00423	C9H8O2	148.05243	Phenylpropanoids	2.84	+
71	Xanthotoxin	298-81-7	C01864	C12H8O4	216.04226	Phenylpropanoids	9.24	+
72	Isobergapten	482-48-4	C18082	C12H8O4	216.04226	Coumarins	9.94	+
73	Sphondin	483-66-9	C18081	C12H8O4	216.04226	Coumarins	8.74	+
74	Guanine	73-40-5	C00242	C5H5N5O	151.04941	Nucleotide and its derivates	2.63	+
75	Methyl anthranilate	134-20-3	C20634	C8H9NO2	151.063329	Alkaloids	2.11	+
76	5-oxoproline	98-79-3	C01879	C5H7NO3	129.042594	Amino acid and derivatives	0.56	+
77	2-(Methylamino)benzoic acid	119-68-6	C03005	C8H9NO2	151.063329	Benzene and substituted derivatives	1.58	+
78	Tryptamine	61-54-1	C00398	C10H12N2	160.100048	Tryptamine derivatives	2.86	+
79	Anatabine	2743-90-0;581-49-7	C10126	C10H12N2	160.100048	Alkaloids	2.82	+
80	Pimelic acid	111-16-0	C02656	C7H12O4	160.07356	Fatty Acyls	0.55	+
81	Tricholomic acid	2644-49-7	C08298	C5H8N2O4	160.048408	Carboxylic acids and derivatives	3.61	+
82	Itaconic acid	97-65-4	C00490	C5H6O4	130.02661	Fatty Acyls	0.52	+
83	2-Phenylacetamide	103-81-1	C02505	C8H9NO	135.068414	Benzene and substituted derivatives	2.52	+
84	Adenine	73-24-5	C00147	C5H5N5	135.054495	Nucleotide and its derivates	2.6	+

85	Terpinolene	586-62-9	C06075	C10H16	136.1252	Monoterpenoids	10.57	+
86	3-Hydroxypicolinic acid	874-24-8	C18620	C6H5NO3	139.026944	Pyridines and derivatives	4.15	+
87	N-Acetyl-L-glutamate 5-semialdehyde	13074-21-0	C01250	C7H11NO4	173.068809	Carboxylic acids and derivatives	1.51	+
88	N-Acetyl-l-leucine	1188-21-2	C02710	C8H15NO3	173.105194	Amino acid and derivatives	5.38	+
89	Xanthotoxol	2009-24-7	C00841	C11H6O4	202.02661	Phenylpropanoids	8.74	+
90	Bergaptol	486-60-2	C00758	C11H6O4	202.02661	Coumarins	7.44	+
91	L-Gulose	6027-89-0	C15923	C6H12O6	180.06339	Carbohydrates	8.71	+
92	4-Sulfobenzoate	636-78-2	C02236	C7H6O5S	201.993596		4.68	+
93	N5-(L-1-Carboxyethyl)-L-ornithine		C04210	C8H16N2O4	204.111008		2.99	+
94	Vasicinol	5081-51-6	C10743	C11H12N2O2	204.089878	Alkaloids	10.57	+
95	Mellein	480-33-1		C10H10O3	178.062995	Coumarins	6.45	+
96	2-Keto-6-acetamidocaproate		C05548	C8H13NO4	187.084459	Keto acids and derivatives	1.48	+
97	Kynurenic acid	492-27-3	C01717	C10H7NO3	189.042594	Alkaloids	4.36	+
98	Ligustilide	4431-01-0	C16987	C12H14O2	190.09938	Miscellaneous	2.8	+
99	Bornyl acetate	5655-61-8	C09837	C12H20O2	196.14633	Monoterpenoids	6.54	+
100	Jasmonic acid	6894-38-8	C08491	C12H18O3	210.125595	Fatty Acyls	6.73	+
101	Harmaline	304-21-2	C06536	C13H14N2O	214.110613	Alkaloids	10	+
102	Flavone	525-82-6	C15608;C10043	C15H10O2	222.06808	Flavonoids	4.13	+
103	Isofraxidin	486-21-5	C17480	C11H10O5	222.052825	Phenylpropanoids	4.25	+
104	2-Benzal-4-hydroxyacetophenone	2657-25-2	C14232	C15H12O2	224.08373	Flavonoids	5.1	+
105	Pogostone	23800-56-8		C12H16O4	224.10486	Miscellaneous	4.95	+
106	2-Deoxyribose 5'-phosphate	102916-66-5	C00673	C5H11O7P	214.024242	Carbohydrates	11.13	+
107	11-Hydroxycanthin-6-one	75969-83-4	C09212	C14H8N2O2	236.058578	Alkaloids	8.8	+
108	Thymidine	50-89-5	C00214	C10H14N2O5	242.090273	Nucleotide and its derivates	3.04	+
109	5'-Deoxyadenosine	4754-39-6	C05198	C10H13N5O3	251.10184	Nucleotide and its derivates	2.41	+
110	Pelargonidin	7690-51-9;134-04-3	C05904	C15H10O5	270.052825	Flavonoids	8.98	+
111	Lucidin	478-08-0	C10369	C15H10O5	270.052825	Anthraquinones	8.89	+
112	6,7,4'-Trihydroxyisoflavone	17817-31-1	C14314	C15H10O5	270.052825	Flavonoids	10.76	+
113	Resokaempferol	2034-65-3	C10037	C15H10O5	270.052825	Flavonoids	8.79	+
114	Pinostrobin	480-37-5	C16419	C16H14O4	270.08921	Flavonoids	8.78	+
115	Naringenin	480-41-1	C00509	C15H12O5	272.068475	Flavonoids	9.03	+
116	Naringenin chalcone	25515-46-2	C06561	C15H12O5	272.068475	Flavonoids	6.54	+
117	Vestitol	35878-41-2	C16225	C16H16O4	272.10486	Flavonoids	6.51	+
118	Butein	487-52-5	C08578	C15H12O5	272.068475	Chalcones	7.07	+
119	Glycinol	69393-95-9	C01263	C15H12O5	272.068475	Flavonoids	6.55	+
120	16-Hydroxy hexadecanoic acid	506-13-8	C18218	C16H32O3	272.235145	Lipids	6.51	+
121	Dehydronuciferine	7630-74-2		C19H19NO2	293.141579	Alkaloids	5.66	+
122	Asp-Phe methyl ester;Aspartame	22839-47-0	C11045	C14H18N2O5	294.121573	Carboxylic acids and derivatives	3.06	+
123	5'-S-Methyl-5'-thioadenosine	2457-80-9	C00170	C11H15N5O3S	297.089561	Nucleotide and its derivates	3.99	+
124	Uracil	66-22-8	C00106	C4H4N2O2	112.027278	Nucleotide and its derivates	1.91	+
125	Palmitoylethanolamide	544-31-0	C16512	C18H37NO2	299.282429	Carboximidic acids and derivatives	12.61	+
126	Oxymorphone	76-41-5	C08019	C17H19NO4	301.131409	Phenanthrenes and derivatives	11.18	+
127	Quercetin	117-39-5	C00389	C15H10O7	302.042655	Flavonoids	8.07	+
128	6-Hydroxykaempferol	4324-55-4	C10068	C15H10O7	302.042655	Flavonoids	6.84	+
129	Shionone	10376-48-4	C17966	C30H50O	426.386165	Triterpenoids	12	+
130	Adenosine 5'-monophosphate	61-19-8	C00020	C10H14N5O7P	347.063087	Nucleotide and its derivates	1.38	+
131	Acetic acid m-cresyl ester	122-46-3		C9H10O2	150.06808	Phenols	7.28	+
132	2-Carboxybenzaldehyde	119-67-5	C03057	C8H6O3	150.031695	Phenols	3.07	+
133	Octadecanamide	124-26-5	C13846	C18H37NO	283.287514	Fatty Acyls	13.02	+
134	Enoxacin	74011-58-8	C06979	C15H17FN4O3	320.128469	Diazanaphthalenes	10.84	+
135	Kirenol	52659-56-0		C20H34O4	338.24571	Diterpenoids	13.16	+
136	Moracin O	123702-97-6		C19H18O5	326.115425	Phenols	9.52	+
137	Docosahexaenoic acid	6217-54-5	C06429	C22H32O2	328.24023	Fatty Acyls	12.6	+
138	Adenosine 2',3'-cyclic phosphate	634-01-5	C02353	C10H12N5O6P	329.052522	Purine nucleotides	1.86	+
139	Dopamine glucuronide	38632-24-5	C03033	C14H19NO8	329.111069	Organooxygen compounds	5.2	+
140	Aurantio-obtusin	67979-25-3	C17670	C17H14O7	330.073955	Anthraquinones	10.28	+
141	Acetylshikonin	24502-78-1	C17413	C18H18O6	330.11034	Quinones	6.39	+
142	ent-16beta,17-dihydroxy-9(11)-kauren-19-oic acid	55483-24-4		C20H30O4	334.21441	Diterpenoids	7.15	+
143	9,10-DHOME	263399-34-4	C14828	C18H34O4	314.24571	Fatty Acyls	13.3	+
144	Platyphylline	480-78-4	C10363	C18H27NO5	337.188924	Alkaloids	9.28	+
145	Sarracine	2492-09-3	C10383	C18H27NO5	337.188924	Alkaloids	13.14	+
146	3alpha,6beta-Ditigloyloxytropan-7beta-ol	7159-86-6		C18H27NO5	337.188924	Alkaloids	13.21	+
147	AICAR	3031-94-5	C04677	C9H15N4O8P	338.062753	Imidazole ribonucleosides and ribonucleotides	13.28	+
148	8-Geranyloxypsoralen	7437-55-0		C21H22O4	338.15181	Coumarins	13.29	+
149	Demethoxycurcumin	22608-11-3	C17742	C20H18O5	338.115425	Phenols	4.91	+
150	3-Carbamyl-1-methylpyridinium (1-Methylnicotinamide)	3106-60-3	C02918	C7H8N2O	136.063663	Pyridines and derivatives	6.36	+
151	Phenyl acetate	122-79-2	C00548	C8H8O2	136.05243	Phenol esters	10.56	+
152	N,N-Dimethyl-1,4-phenylenediamine	99-98-9	C04203	C8H12N2	136.100048		2.56	+
153	2-Methylbenzoic acid	118-90-1	C07215	C8H8O2	136.05243	Benzene and substituted derivatives	8.83	+
154	4',7-Dihydroxyflavone	2196-14-7	C12123	C15H10O4	254.05791	Flavonoids	7.69	+
155	Chalepensin	13164-03-9	C09165	C16H14O3	254.094295	Coumarins	7.62	+
156	Erucic acid	112-86-7	C08316	C22H42O2	338.31848	Fatty Acyls	10.46	+
157	Galactinol	3687-64-7	C01235	C12H22O11	342.116215	Organooxygen compounds	2.79	+
158	1-O-Caffeoylglucose	14364-08-0	C10433	C15H18O9	342.095085	Carbohydrates	4.92	+
159	Trioxilin A3		C14809	C20H34O5	354.240625	Fatty Acyls	10.97	+
160	Scopolin	531-44-2	C01527	C16H18O9	354.095085	Phenylpropanoids	9.99	+
161	Corynanthine	483-10-3		C21H26N2O3	354.194343	Alkaloids	4.46	+
162	Rosmarinic acid	537-15-5;20283-92-5	C01850	C18H16O8	360.08452	Phenylpropanoids	6.99	+
163	S-Lactoylglutathione	41656-56-8	C03451	C13H21N3O8S	379.104938	Carboxylic acids and derivatives	4.09	+
164	Benzoic acid	65-85-0	C00180	C7H6O2	122.03678	Phenols	5.95	+
165	1-Phenylethanol	98-85-1	C07112	C8H10O	122.073165	Phenols	10.56	+
166	Astrocasine	2114-92-3	C10130	C20H26N2O	310.204513	Alkaloids	13.94	+
167	Moracin C	69120-06-5		C19H18O4	310.12051	Phenols	13.99	+
168	cis-Aconitic acid	499-12-7;585-84-2	C00417	C6H6O6	174.01644	Organic acids and derivatives	1.46	+
169	Juglone	481-39-0	C03840	C10H6O3	174.031695	Quinones	8.74	+
170	Gramine	87-52-5	C08304	C11H14N2	174.115698	Alkaloids	8.73	+

171	Traumatic acid	6402-36-4	C16308	C12H20O4	228.13616	Fatty Acyls	10.49	+
172	Xanthyletin	553-19-5	C09317	C14H12O3	228.078645	Coumarins	8.37	+
173	Acanthoside B	7374-79-0	C10890	C28H36O13	580.215595	Lignans	6.51	+
174	Androstanediol	25126-76-5	C07632	C19H32O2	292.24023	Steroids and steroid derivatives	10.57	+
175	Prostaglandin I2	35121-78-9	C01312	C20H32O5	352.224975	Fatty Acyls	9.39	+
176	Moschamine	68573-23-9		C20H20N2O4	352.142308	Alkaloids	11.11	+
177	Ajmalicine	483-04-5	C09024	C21H24N2O3	352.178693	Alkaloids	7.62	+
178	Savinin	493-95-8	C10880	C20H16O6	352.09469	Lignans	11.11	+
179	Perakine	4382-56-3	C19932	C21H22N2O3	350.163043	Alkaloids	7.19	+
180	Geniposide	24512-63-8	C09781	C17H24O10	388.13695	Iridoids	5.14	+
181	5-Hydroxy-L-tryptophan	4350-09-8	C00643	C11H12N2O3	220.084793	Amino acid and derivatives	10.9	+
182	Allicin	539-86-6	C07600	C6H10OS2	162.017307	Miscellaneous	10.57	+
183	4-Methylumbelliferyl acetate	2747-05-9	C03837	C12H10O4	218.05791	Coumarins	10.06	+
184	Prephenate	126-49-8	C00254	C10H10O6	226.04774	Keto acids and derivatives	2.24	+
185	Genipin	6902-77-8	C09780	C11H14O5	226.084125	Iridoids	4.85	+
186	3-Nitro-L-tyrosine	621-44-3		C9H10N2O5	226.058973	Miscellaneous	8.73	+
187	Deoxylapachol	3568-90-9	C10325	C15H14O2	226.09938	Quinones	4.93	+
188	Glycerophosphocholine	28319-77-9	C00670	C8H20NO6P	257.102826	Cholines	12.87	+
189	Glucose 1-phosphate	59-56-3	C00103	C6H13O9P	260.029722	Organooxygen compounds	13	+
190	Mannose 6-phosphate		C00275	C6H13O9P	260.029722	Organooxygen compounds	13.03	+
191	Knightinol	77053-06-6	C10859	C17H23NO3	289.167794	Alkaloids	5.96	+
192	3-O-Acetylpinobanksin	52117-69-8	C16418	C17H14O6	314.07904	Flavonoids	10.21	+
193	Syringol	91-10-1	C10787	C8H10O3	154.062995	Phenols	4.95	+
194	1,4-Cineole	470-67-7	C16909	C10H18O	154.135765	Monoterpenoids	3.37	+
195	Glycitin	40246-10-4	C16195	C22H22O10	446.1213	Flavonoids	5.7	+
196	Oleamide	301-02-0	C19670	C18H35NO	281.271864	Fatty Acyls	12.54	+
197	Didrovaltrate	18296-45-2	C09776	C22H32O8	424.20972	Iridoids	10.28	+
198	Beta-mangostin	20931-37-7		C25H28O6	424.18859	Xanthones	13.21	+
199	Harpagoside	19210-12-9	C09783	C24H30O11	494.178815	Iridoids	6.59	+
200	Acetyl tryptophan	2280-01-5	C03137	C13H14N2O3	246.100443	Amino acid and derivatives	7.97	+
201	Hypaphorine	487-58-1	C09213	C14H18N2O2	246.136828	Alkaloids	8.37	+
202	Xanthatin	26791-73-1	C09601	C15H18O3	246.125595	Sesquiterpenoids	8.39	+
203	Curcolone	17015-43-9		C15H18O3	246.125595	Sesquiterpenoids	8.37	+
204	3-Galloylquinic acid	17365-11-6	C10834	C14H16O10	344.07435	Phenols	2.9	+
205	Diosbulbin D	66756-57-8		C19H20O6	344.12599	Diterpenoids	2.63	+
206	Chamazulene	529-05-5	C09633	C14H16	184.1252	lipids	8.74	+
207	Methyl gallate	99-24-1		C8H8O5	184.037175	Phenols	4.44	+
208	cis-3-(Carboxy-ethyl)-3,5-cyclo-hexadiene-1,2-diol		C11588	C9H12O4	184.07356		4.85	+
209	Chelidonic acid	99-32-1	C08476	C7H4O6	184.00079	Miscellaneous	1.39	+
210	(S)-2-Acetolactate		C06010	C5H8O4	132.04226	Keto acids and derivatives	1.32	+
211	Cinnamaldehyde	14371-10-9;104-55-2	C00903	C9H8O	132.057515	Phenylpropanoids	6.34	+
212	Atropaldehyde		C16592	C9H8O	132.057515	Benzene and substituted derivatives	6.34	+
213	Ginkgolide A	15291-75-5	C07601	C20H24O9	408.142035	Diterpenoids	8.27	+
214	Ginkgolide C	15291-76-6	C07603	C20H24O11	440.131865	Diterpenoids	10.47	+
215	Liriodendrin	573-44-4		C34H46O18	742.26842	Lignans	12.13	+
216	Capsanthin	465-42-9	C08584	C40H56O3	584.422945	Terpene	12.8	+
217	Lutein	127-40-2	C08601	C40H56O2	568.42803	Prenol lipids	13.14	+
218	Artemisinin	63968-64-9	C09538	C15H22O5	282.146725	Sesquiterpenoids	13.02	+
219	Octyl Gallate	1034-01-1		C15H22O5	282.146725	Phenols	12.97	+
220	Aloperine	56293-29-9	C10748	C15H24N2	232.193948	Alkaloids	7.22	+
221	Dihydrokavain	587-63-3		C14H16O3	232.109945	Phenols	10.42	+
222	Cryptomeridiol 11-rhamnoside	349112-30-7		C21H38O6	386.26684	Sesquiterpenoids	8.31	+
223	Nandrolone	434-22-0	C07254	C18H26O2	274.19328	Steroids and steroid derivatives	10.58	+
224	(-)-Epiafzelechin	24808-04-6		C15H14O5	274.084125	Flavonoids	5.66	+
225	Metanephrine	5001-33-2	C05588	C10H15NO3	197.105194	Phenols	2.13	+
226	Cyclomusalenone	30452-60-9		C30H48O	424.370515	Triterpenoids	9.42	+
227	Taraxasterone	6786-16-9		C30H48O	424.370515	Triterpenoids	13.85	+
228	(-)-Salsoline	89-31-6	C09640	C11H15NO2	193.110279	Tetrahydroisoquinolines	4.25	+
229	Gentioflavin	18058-50-9	C09962	C10H11NO3	193.073894	Alkaloids	4.93	+
230	Phytic acid	83-86-3	C01204	C6H18O24P6	659.861382	Miscellaneous	4.47	+
231	Mirtazapine	61337-67-5;85650-52-8	C07570	C17H19N3	265.157897	Piperazinoazepines	8.77	+
232	(-)-Anonaine	1862-41-5	C09339	C17H15NO2	265.110279	Alkaloids	7.42	+
233	Caffeine	58-08-2	C07481	C8H10N4O2	194.080376	Alkaloids	4.5	+
234	Ferulic acid; Trans-Ferulic acid;trans-Ferulic acid	1135-24-6;537-98-4	C01494	C10H10O4	194.05791	Phenylpropanoids	4.86	+
235	5-Hydroxyconiferaldehyde	249647-14-1	C12204	C10H10O4	194.05791		4.61	+
236	Kakuol	18607-90-4	C16982	C10H10O4	194.05791	Phenols	4.58	+
237	Butyl 4-Hydroxybenzoate	94-26-8		C11H14O3	194.094295	Lignans	4.56	+
238	Lycorine	476-28-8	C08532	C16H17NO4	287.115759	Alkaloids	7.59	+
239	Pipermethystine	71627-22-0		C16H17NO4	287.115759	Alkaloids	7.57	+
240	Osajin	482-53-1	C10511	C25H24O5	404.162375	Flavonoids	11.06	+
241	Mangiferin	4773-96-0	C10077	C19H18O11	422.084915	Xanthones	5.14	+
242	Lamiide	27856-54-8	C11644	C17H26O12	422.14243	Iridoids	10.74	+
243	Lucidadiol	252351-95-4		C30H48O3	456.360345	Triterpenoids	12.64	+
244	Kazinol A	99624-28-9	C09760	C25H30O4	394.21441	Flavonoids	13.26	+
245	Rotenone	83-79-4	C07593	C23H22O6	394.14164	Flavonoids	12.92	+
246	Deguelin	522-17-8	C10417	C23H22O6	394.14164	Flavonoids	13.46	+
247	Theobromine	83-67-0	C07480	C7H8N4O2	180.064726	Alkaloids	3.27	+
248	Caffeic acid	331-39-5	C01481	C9H8O4	180.04226	Phenylpropanoids	4.44	+
249	Coniferyl alcohol	458-35-5;32811-40-8	C00590	C10H12O3	180.078645	Phenylpropanoids	3.27	+
250	2-Hydroxy-3-(4-hydroxyphenyl)propenoic acid		C05350	C9H8O4	180.04226	Benzene and substituted derivatives	10.05	+
251	Propyl paraben	94-13-3		C10H12O3	180.078645	Phenols	3.26	+
252	Neosperidin dihydrochalcone	20702-77-6		C28H36O15	612.205425	Chalcones	12.46	+
253	Hydroxysafflor yellow A	78281-02-4		C27H32O16	612.16904	Chalcones	12.59	+
254	Quercitrin	522-12-3	C01750	C21H20O11	448.100565	Flavonoids	6.85	+
255	Luteolin-7-O-glucoside;2-(3,4-dihydroxyphenyl)-5-hydroxy-7-[(2S,3R,4S,5S,6R)<br>-3,4,5-trihydroxy-6-(hydroxymethyl)oxan-2-yl]oxychromen-4-one	5373-11-5	C03951	C21H20O11	448.100565	Flavonoids	6.1	+

256	Methylecgonine	7143-09-1	C12448	C10H17NO3	199.120844	Alkaloids	3.14	+
257	Tussilagine	80151-77-5	C10411	C10H17NO3	199.120844	Alkaloids	3.85	+
258	1,2,3-Tri-n-Octanoylglycerol	538-23-8	C13044	C27H50O6	470.36074	Miscellaneous	10.57	+
259	Withaferin A	5119-48-2	C08841	C28H38O6	470.26684	Steroids and steroid derivatives	10.57	+
260	Cymarin	508-77-0	C08859	C30H44O9	548.298535	Steroids and steroid derivatives	9.11	+
261	Esculin	531-75-9	C09264	C15H16O9	340.079435	Coumarins	4.23	+
262	Trimethylapigenin	5631-70-9		C18H16O5	312.099775	Flavonoids	10.8	+
263	Broussonin C	76045-49-3	C09524	C20H24O3	312.172545	Phenols	13.23	+
264	Desacetylmatricarin	10180-88-8		C15H18O4	262.12051	Sesquiterpenoids	8.73	+
265	Maclurin	519-34-6	C09951	C13H10O6	262.04774	Xanthones	8.73	+
266	2-Hydroxy-6-oxo-6-(2-carboxyphenyl)-hexa-2,4-dienoate		C16264	C13H10O6	262.04774		8.73	+
267	Pristimerin	1258-84-0	C08633	C30H40O4	464.29266	Triterpenoids	6.85	+
268	indolin-2-one	59-48-3	C12312	C8H7NO	133.052764	Alkaloids	5.67	+
269	Cephalotaxine	24316-19-6	C10580	C18H21NO4	315.147059	Alkaloids	4.93	+
270	Rosmarinine	520-65-0	C10380	C18H27NO6	353.183839	Alkaloids	9.38	+
271	Boldine	476-70-0	C09365	C19H21NO4	327.147059	Alkaloids	8.59	+
272	Scoulerine	6451-73-6	C02106	C19H21NO4	327.147059	Alkaloids	5.77	+
273	Agrocybenine	178764-92-6		C12H18N2O	206.141913	Alkaloids	5.03	+
274	Ethyl trans-p-methoxycinnamate	1929-30-2	C10476	C12H14O3	206.094295	Cinnamic acids and derivatives	4.97	+
275	2-Hydroxy-3-methylbenzalpyruvate		C14086	C11H10O4	206.05791		5.07	+
276	3-Methoxy-4,5-methylenedioxycinnamaldehyde	74683-19-5		C11H10O4	206.05791	Phenylpropanoids	5.04	+
277	Narciclasine	29477-83-6	C08533	C14H13NO7	307.069204	Alkaloids	4.12	+
278	Rutacridone	17948-33-3	C10738	C19H17NO3	307.120844	Alkaloids	12.87	+
279	Crategolic acid	4373-41-5	C16939	C30H48O4	472.35526	Triterpenoids	12.48	+
280	Alphitolic acid	19533-92-7	C16912	C30H48O4	472.35526	Triterpenoids	10.56	+
281	N1-Methyl-4-pyridone-3-carboxamide	769-49-3	C05843	C7H8N2O2	152.058578	Pyridines and derivatives	2.66	+
282	3,4-Dihydroxyphenylglycol	28822-73-3;3343-19-9	C05576	C8H10O4	170.05791	Phenols	3.08	+
283	3-Hydroxyphenylacetic acid	621-37-4	C05593	C8H8O3	152.047345	Phenols	5.06	+
284	4-(Ethoxymethyl)phenol	57726-26-8		C9H12O2	152.08373	Phenols	2.63	+
285	(+)-Camphor	464-49-3	C00808	C10H16O	152.120115	Monoterpenoids	6.5	+
286	3-(4-Hydroxyphenyl)-1-propanol	10210-17-0		C9H12O2	152.08373	Phenylpropanoids	3.09	+
287	3,4-Dihydroxyphenylacetaldehyde	5707-55-1	C04043	C8H8O3	152.047345	Benzene and substituted derivatives	4.05	+
288	(+)-Pteryxin	17944-23-9;13161-75-6	C09307	C21H22O7	386.136555	Coumarins	11.11	+
289	Samidin	477-33-8	C09310	C21H22O7	386.136555	Coumarins	5.94	+
290	Cleomiscosin A	76948-72-6	C09922	C20H18O8	386.10017	Coumarins	5.74	+
291	Pinoresinol dimethyl ether	29106-36-3	C10561	C22H26O6	386.17294	Lignans	5.15	+
292	trans-Hinokiresinol	17676-24-3	C10628	C17H16O2	252.11503	Lignans	10.62	+
293	Morusin	62596-29-6	C10106	C25H24O6	420.15729	Flavonoids	4.9	+
294	Cheilanthifoline	483-44-3	C05174	C19H19NO4	325.131409	Miscellaneous	14.13	+
295	Cassythicine	5890-28-8	C09389	C19H19NO4	325.131409	Alkaloids	9.9	+
296	L-Pipecolic acid	535-75-1;3105-95-1	C00408	C6H11NO2	129.078979	Amino acid and derivatives	0.69	+
297	Pyrrolidonecarboxylic acid	4042-36-8	C02237	C5H7NO3	129.042594	Carboxylic acids and derivatives	0.67	+
298	Deltaline	3836-11-9;6836-11-9	C08679	C27H41NO8	507.283219	Alkaloids	7.55	+
299	Morroniside	25406-64-8	C17000	C17H26O11	406.147515	Iridoids	4.49	+
300	Vitexin 2''-glucoside	61360-94-9	C04024	C27H30O15	594.158475	Flavonoids	4.91	+
301	Saponarin	20310-89-8	C08064	C27H30O15	594.158475	Flavonoids	4.94	+
302	Lonicerin	25694-72-8	C12630	C27H30O15	594.158475	Flavonoids	6.08	+
303	Poncirin	14941-08-3	C09830	C28H34O14	594.19486	Flavonoids	8.16	+
304	Chrysoeriol 7-apiosylglucoside	33579-63-4		C27H30O15	594.158475	Flavonoids	5.32	+
305	Lactupicrin	65725-11-3	C09490	C23H22O7	410.136555	Sesquiterpenoids	13.8	+
306	alpha-Hederin	27013-91-8	C08954	C41H66O12	750.45543	Triterpenoids	8.16	+
307	Kaempferitrin	482-38-2	C16981	C27H30O14	578.16356	Flavonoids	5.69	+
308	Daidzein-4',7-diglucoside	53681-67-7		C27H30O14	578.16356	Flavonoids	5.94	+
309	Desoxypeganine	495-59-0	C10656	C11H12N2	172.100048	Alkaloids	8.38	+
310	Nobiletin	478-01-3	C10112	C21H22O8	402.13147	Flavonoids	11.03	+
311	Veraguensin	19950-55-1	C10892	C22H28O5	372.193675	Lignans	6.75	+
312	Loganin	18524-94-2	C01433	C17H26O10	390.1526	Terpene	5.1	+
313	Cyclocurcumin	153127-42-5		C21H20O6	368.12599	Phenols	11.18	+
314	C-Veratroylglycol	168293-10-5		C10H12O5	212.068475	Phenylpropanoids	3.47	+
315	Atranorin	479-20-9		C19H18O8	374.10017	Phenols	5.26	+
316	Vitexicarpin	479-91-4		C19H18O8	374.10017	Flavonoids	10.98	+
317	Geniposidic acid;Geniposidic acid	27741-01-1	C11673	C16H22O10	374.1213	Iridoids;Terpene	3.7	+
318	8-Hydroxypinoresinol	81426-17-7		C20H22O7	374.136555	Lignans	8.46	+
319	Methyl rosmarinate	99353-00-1		C19H18O8	374.10017	Phenylpropanoids	6.6	+
320	Schaftoside	51938-32-0	C10181	C26H28O14	564.14791	Flavonoids	5.39	+
321	Isovitexin 2''-O-arabinoside	53382-71-1		C26H28O14	564.14791	Flavonoids	5.25	+
322	Carbofuran	1563-66-2	C14291	C12H15NO3	221.105194	Coumarans	4.03	+
323	Dihydrozeatin	23599-75-9	C02029	C10H15N5O	221.12766	Imidazopyrimidines	4.3	+
324	Purine	120-73-0	C15587	C5H4N4	120.043596	Nucleotide and its derivates	2.05	+
325	Erythrose	583-50-6	C01796	C4H8O4	120.04226	Organooxygen compounds	3.05	+
326	Styrene Oxide	96-09-3	C02083	C8H8O	120.057515	Benzene and substituted derivatives	5.94	+
327	Isoxanthopterin	529-69-1	C03975	C6H5N5O2	179.044325	Pteridines and derivatives	3.26	+
328	Glucosamine	3416-24-8	C00329	C6H13NO5	179.079374	Carbohydrates	2.6	+
329	Sinapyl alcohol	537-33-7	C02325	C11H14O4	210.08921	Hydroxycinnamoyl derivatives	4.43	+
330	Pelargonidin-3-O-glucoside	18466-51-8	C12137	C21H20O10	432.10565	Flavonoids	5.94	+
331	Vitexin	3681-93-4	C01460	C21H20O10	432.10565	Flavonoids	5.92	+
332	Afzelin;Kaempferol 3-O-rhamnoside (Kaempferin)	482-39-3	C16911	C21H20O10	432.10565	Flavonoids	5.95	+
333	Glyceric acid	473-81-4	C00258	C3H6O4	106.02661	Organooxygen compounds	0.55	+
334	L-Hypoglycin A	156-56-9	C08287	C7H11NO2	141.078979	Carboxylic acids and derivatives	0.52	+
335	Cupressuflavone	3952-18-9	C10034	C30H18O10	538.09	Flavonoids	9.67	+
336	Lithospermic acid	28831-65-4	C08745	C27H22O12	538.11113	Phenylpropanoids	7.12	+
337	Myricitrin;Myricetin 3-O-rhamnoside (Myricitrin)	17912-87-7	C10108	C21H20O12	464.09548	Flavonoids	6.84	+
338	Isoquercitrin	21637-25-2	C05623	C21H20O12	464.09548	Flavonoids	6.22	+
339	4-Hydroxyglucobrassicin	83327-20-2	C08422	C16H20N2O10S2	464.05594	Miscellaneous	6.07	+
340	Isoliquiritin; Isoliquiritoside	5041-81-6	C16978	C21H22O9	418.126385	Chalcones	6.94	+
341	Cryptomeridiol	4666-84-6	C17676	C15H28O2	240.20893	Sesquiterpenoids	3.24	+

342	Ethyl 3,4,5-trimethoxybenzoate	6178-44-5		C12H16O5	240.099775	Phenols	0.53	+
343	Salannin	992-20-1	C08780	C34H44O9	596.298535	Triterpenoids	5.97	+
344	Isoacteoside	61303-13-7		C29H36O15	624.205425	Phenylpropanoids	6.63	+
345	Grossamide	80510-06-1		C36H36N2O8	624.247168	Alkaloids	13.08	+
346	Plantagoside	78708-33-5	C17531	C21H22O12	466.11113	Flavonoids	6.85	+
347	Agnuside	11027-63-7	C09765	C22H26O11	466.147515	Iridoids	12.37	+
348	Curcumenone	100347-96-4	C17492	C15H22O2	234.16198	Sesquiterpenoids	10.56	+
349	Sparteine	90-39-1	C10783	C15H26N2	234.209598	Alkaloids	8.06	+
350	Angustifoline	550-43-6	C10751	C14H22N2O	234.173213	Alkaloids	10.56	+
351	beta-Costic acid	3650-43-9		C15H22O2	234.16198	Sesquiterpenoids	8.9	+
352	Mulberrofuran A	68978-04-1	C08846	C25H28O4	392.19876	Phenols	13.23	+
353	7-Ethyl-10-Hydroxycamptothecin	86639-52-3	C11173	C22H20N2O5	392.137223	Alkaloids	6.28	+
354	5-Hydroxymethyluracil	4433-40-3	C03088	C5H6N2O3	142.037843	Nucleotide and its derivates	2.79	+
355	Vanillic acid	121-34-6	C06672	C8H8O4	168.04226	Phenols	3.78	+
356	Plumbagin	481-42-5	C10387	C11H8O3	188.047345	Quinones	8.73	+
357	Vasicine	6159-55-3	C10733	C11H12N2O	188.094963	Alkaloids	3.76	+
358	2,6-Dimethyl-7-octene-2,3,6-triol	73815-21-1		C10H20O3	188.141245	Monoterpenoids	3.82	+
359	Harman	486-84-0	C09209	C12H10N2	182.084398	Alkaloids	8.6	+
360	Dihydroconiferyl alcohol	2305-13-7	C10448	C10H14O3	182.094295	Phenylpropanoids	7.99	+
361	Leucodopachrome	18766-67-1	C05604	C9H9NO4	195.053159	Indoles and derivatives	4.82	+
362	Guaiacol	90-05-1	C15572;C01502	C7H8O2	124.05243	Phenols	5.62	+
363	9-Riburonosyladenine		C11501	C10H11N5O5	281.07602		12.54	+
364	4-Hydroxyphenylacetylglutamic acid		C05595	C13H15NO6	281.089939		12.99	+
365	Xanthurenic acid	59-00-7	C02470	C10H7NO4	205.037509	Quinolines and derivatives	2.96	+
366	10-Formyldihydrofolate	28459-40-7	C03204	C20H21N7O7	471.150248	Pteridines and derivatives	10.56	+
367	Nicotinate ribonucleoside		C05841	C11H13NO6	255.074289	Nicotinic acid derivatives	4.55	+
368	N-D-Glucosylarylamine		C03142	C12H17NO5	255.110674		2.69	+
369	Tetrahydroepiberberine	38853-67-7	C17898	C20H21NO4	339.147059	Alkaloids	8.74	+
370	Dulcoside A	64432-06-0		C38H60O17	788.383055	Diterpenoids	7.54	+
371	DL-Tyrosine	556-03-6	C01536	C9H11NO3	181.073894	Alkaloids	1.9	+
372	Terpinine-4-ol	562-74-3	C17073	C10H18O	154.135765	Monoterpenoids	5.45	+
373	Sinapine	18696-26-9	C00933	C16H23NO5	309.157624	Cholines	5.08	+
374	Fulvine	6029-87-4	C10304	C16H23NO5	309.157624	Alkaloids	7.99	+
375	Palmitic acid	57-10-3	C00249	C16H32O2	256.24023	Lipids	12.92	+
376	Huperzine B	103548-82-9	C09866	C16H20N2O	256.157563	Alkaloids	3.83	+
377	Oleuropein	32619-42-4	C09794	C25H32O13	540.184295	Iridoids	7.4	+
378	N,N-Dimethylaniline	121-69-7	C02846	C8H11N	121.089149	Organonitrogen compounds	2.74	+
379	4-Methyl-5-thiazoleethanol	137-00-8	C04294	C6H9NOS	143.040485	Azoles	2.66	+
380	Eicosadienoic acid	2091-39-6	C16525	C20H36O2	308.27153	Fatty Acyls	10.62	+
381	2-Biphenylol	90-43-7	C02499	C12H10O	170.073165	Benzene and substituted derivatives	8.73	+
382	1,2-Dihydroxy-6-methylcyclohexa-3,5-dienecarboxylate		C06731	C8H10O4	170.05791		4.2	+
383	2-(2-Hydroxy-2-propyl)-5-methyl-5-vinyltetrahydrofuran	60047-17-8		C10H18O2	170.13068	Monoterpenoids	5.47	+
384	Furfuryl acetate	623-17-6		C7H8O3	140.047345	Miscellaneous	5.47	+
385	Dhurrin	499-20-7	C05143	C14H17NO7	311.100504	Alkaloids	13.33	+
386	Dihydrosanguinarine	3606-45-9	C05191	C20H15NO4	333.100109	Alkaloids	7.62	+
387	Norchelerythrine	6900-99-8	C12226	C20H15NO4	333.100109	Alkaloids	7.47	+
388	Amygdalin	29883-15-6	C08325	C20H27NO11	457.158414	Phenols	4.88	+
389	Stearic Acid	57-11-4	C01530	C18H36O2	284.27153	Fatty Acyls	13.02	+
390	Taxiphyllin	21401-21-8	C01855	C14H17NO7	311.100504	Phenols	13.76	+
391	Daidzin	552-66-9	C10216	C21H20O9	416.110735	Flavonoids	5.57	+
392	Ganoderic acid D2	97653-94-6		C30H42O8	530.28797	Triterpenoids	12.6	+
393	Coclaurine	486-39-5	C06161	C17H19NO3	285.136494	Alkaloids	9.06	+
394	(S)-N-Methylcoclaurine	3423-07-2	C05176	C18H21NO3	299.152144	Isoquinolines and derivatives	7.59	+
395	Magnoflorine	2141-09-5	C09581	C20H23NO4	341.162709	Alkaloids	6.55	+
396	Rotundine	10097-84-4;483-14-7	C02890	C21H25NO4	355.178359	Alkaloids	10.96	+
397	(S)-Actinidine	524-03-8	C09910	C10H13N	147.104799	Pyridines and derivatives	2.8	+
398	1H-Indole-2,3-dione	91-56-5	C11129	C8H5NO2	147.032029	Indoles and derivatives	2.79	+
399	Ecgonine	481-37-8	C10858	C9H15NO3	185.105194	Alkaloids	3.11	+
400	Otonecine	6887-34-9	C10356	C9H15NO3	185.105194	Alkaloids	2.97	+
401	Piperidine	110-89-4	C01746	C5H11N	85.089149	Alkaloids	2.25	+
402	2-Hydroxypyridine	142-08-5	C02502	C5H5NO	95.037114	Alkaloids	3.58	+
403	3-Methyl-1-butylamine	107-85-7	C02640	C5H13N	87.104799	Alkaloids	3.59	+
404	Dexmedetomidine	113775-47-6	C07450	C13H16N2	200.131348	Benzene and substituted derivatives	10.51	+
405	4,4'-Methylenediphenol	620-92-8	C14298	C13H12O2	200.08373	Phenols	8.73	+
406	2-Hydroxy-6-oxo-(2'-aminophenyl)-hexa-2,4-dienoate		C08062	C12H11NO4	233.068809		10.57	+
407	Glucoiberin	554-88-1	C08411	C11H21NO10S3	423.032762	Organooxygen compounds	10.47	+
408	7-(4-Hydroxyphenyl)-1-phenyl-4-hepten-3-one	100667-52-5		C19H20O2	280.14633	Phenols	12.53	+
409	Guanosine	118-00-3	C00387	C10H13N5O5	283.09167	Nucleotide and its derivates	2.65	+
410	Colchicine	64-86-8	C07592	C22H25NO6	399.168189	Alkaloids	5.09	+
411	Uplandicine	74202-10-1	C10412	C17H27NO7	357.178754	Alkaloids	3.47	+
412	Talatisamine	20501-56-8	C08713	C24H39NO5	421.282824	Alkaloids	4.66	+
413	2,3-Dehydrosilybin A	25166-14-7		C25H20O10	480.10565	Flavonoids	5.53	+
414	Helveticoside	630-64-8	C08869	C29H42O9	534.282885	Steroids and steroid derivatives	6.5	+
415	Ganoderic acid L	102607-24-9		C30H46O8	534.31927	Alkaloids	5.82	+
416	Phillyrin;Phillyroside	487-41-2	C17048	C27H34O11	534.210115	Phenylpropanoids	7.76	+
417	5-Tricosyl-1,3-benzenediol	70110-60-0		C29H52O2	432.39673	Phenols	13.85	+
418	Sarmentosin	71933-54-5	C08340	C11H17NO7	275.100504	Miscellaneous	10.56	+
419	Ginsenoside Rg1	22427-39-0	C08946	C42H72O14	800.49221	Triterpenoids	7.9	+
420	1,3-Diphenyl-2-propen-1-one;Chalcone	94-41-7	C01484	C15H12O	208.088815	Flavonoids	8.25	+
421	Fraxetin	574-84-5	C09265	C10H8O5	208.037175	Coumarins	5.26	+
422	trans-3,5-Dimethoxy-4-hydroxy cinnamaldehydee	4206-58-0	C05610	C11H12O4	208.07356	Phenylpropanoids	5.17	+
423	DL-Benzylsuccinic acid		C09816	C11H12O4	208.07356	Phenylpropanoids	4.95	+
424	Primin	15121-94-5	C10390	C12H16O3	208.109945	Quinones	5.17	+
425	Actinidic acid	341971-45-7		C30H46O5	486.334525	Triterpenoids	12.94	+
426	Soyasaponin I	51330-27-9	C08983	C48H78O18	942.51882	Triterpenoids	9.53	+
427	22-Dehydroclerosterol	26315-07-1		C29H46O	410.354865	Steroids	12.59	+

428	Angelicin	523-50-2	C09060	C11H6O3	186.031695	Phenylpropanoids	8.97	+
429	Deoxyvasicinone	530-53-0	C10659	C11H10N2O	186.079313	Alkaloids	10.57	+
430	1-Naphthylacetic acid	86-87-3	C13014	C12H10O2	186.06808	Phytohormones	10.55	+
431	Ergocristine	511-08-0	C09164	C35H39N5O5	609.29512	Alkaloids	9.18	+
432	Fraxin	524-30-1	C09266	C16H18O10	370.09	Coumarins	9.01	+
433	Ipecoside	15401-60-2	C09464	C27H35NO12	565.215929	Iridoids	12.7	+
434	Neoastilbin	54081-47-9	C09803	C21H22O11	450.116215	Flavonoids	5.97	+
435	Phytanic acid	14721-66-5	C01607	C20H40O2	312.30283	Lipids	11.2	+
436	Procyanidin A2	41743-41-3	C10237	C30H24O12	576.12678	Flavonoids	8.38	+
437	Rivularine	723-78-4	C10278	C13H19NO3	237.136494	Alkaloids	4.35	+
438	Crotanecine	5096-50-4	C10284	C8H13NO3	171.089544	Alkaloids	4.2	+
439	Petasitenine	60102-37-6	C10359	C19H27NO7	381.178754	Alkaloids	4.68	+
440	Phalaenopsine T	23412-97-7	C10361	C20H27NO5	361.188924	Alkaloids	10.54	+
441	Cardanol (C15:1)	501-26-8	C10785	C21H34O	302.260965	Phenols	12.69	+
442	Jervine	469-59-0	C10811	C27H39NO3	425.292994	Alkaloids	10.52	+
443	Beta-Solamarine	3671-38-3		C45H73NO15	867.498024	Alkaloids	7.65	+
444	Solamarine	20318-30-3		C45H73NO16	883.492939	Alkaloids	7.68	+
445	Apoatropine	500-55-0	C10843	C17H21NO2	271.157229	Alkaloids	8.78	+
446	Cochlearine	52418-07-2	C10853	C15H19NO3	261.136494	Alkaloids	13.76	+
447	Benzofuran	271-89-6	C14512	C8H6O	118.041865	Benzofurans	5.15	+
448	Genipin-1-O-gentiobioside	29307-60-6	C16965	C23H34O15	550.189775	Iridoids	4.67	+
449	Yatein	40456-50-6	C10557	C22H24O7	400.152205	Lignans	12.6	+
450	10-Gingerol	23513-15-7	C17496	C21H34O4	350.24571	Phenols	12.44	+
451	Ginsenoside Ro	34367-04-9	C17543	C48H76O19	956.498085	Triterpenoids	9.58	+
452	Ethyl acrylate	140-88-5	C19238	C5H8O2	100.05243	Carboxylic acids and derivatives	1.74	+
453	N-Nitroso-pyrrolidine	930-55-2	C19285	C4H8N2O	100.063663	Pyrrolidines	3.11	+
454	Tuberostemonine	6879-01-2		C22H33NO4	375.240959	Alkaloids	6.51	+
455	Methylisopelletierine	18747-42-7	C06184	C9H17NO	155.131014	Alkaloids	4.11	+
456	Retronecine	480-85-3	C06177	C8H13NO2	155.094629	Alkaloids	3	+
457	1-Isomangostin hydrate	26063-95-6		C24H28O7	428.183505	Xanthones	13.5	+
458	Roemerine	548-08-3		C18H17NO2	279.125929	Alkaloids	8.09	+
459	3-Hydroxy-2-methylpyridine	1121-25-1		C6H7NO	109.052764	Alkaloids	1.44	+
460	Salviaflaside	178895-25-5		C24H26O13	522.137345	Phenylpropanoids	7.12	+
461	Ganoderenic acid E	110241-23-1		C30H40O8	528.27232	Triterpenoids	12.79	+
462	Vitamin A	68-26-8;11103-57-4	C00473	C20H30O	286.229665	Vitamins	9.86	+
463	Formononetin	485-72-3	C00858	C16H12O4	268.07356	Flavonoids	9.94	+
464	Tectochrysin	520-28-5	C11621	C16H12O4	268.07356	Flavonoids	9.88	+
465	Inosine	58-63-9	C00294	C10H12N4O5	268.080771	Nucleotide and its derivates	2.6	+
466	Ginsenoside Rd	52705-93-8	C20725	C48H82O18	946.55012	Triterpenoids	10.08	+
467	Ganoderic acid S	104759-35-5		C30H44O3	452.329045	Triterpenoids	9.21	+
468	Malvidin 3,5-diglucoside (Malvin)	16727-30-3	C08718	C29H35O17	655.18743	Flavonoids	12.52	+
469	Cassiaside B	119170-51-3		C26H30O14	566.16356	Phenols	13.12	+
470	Soyasaponin IV	108906-97-4		C41H66O13	766.450345	Triterpenoids	10.25	+
471	Ginsenoside F1	53963-43-2		C36H62O9	638.439385	Triterpenoids	9.11	+
472	Macamide B	74058-71-2		C23H39NO	345.303164	Alkaloids	8.85	+
473	Buddlenoid A	142750-32-1		C30H26O13	594.137345	Flavonoids	8.17	+
474	Raloxifene	82640-04-8;84449-90-1	C07228	C28H27NO4S	473.16608	Organooxygen compounds	8.91	+
475	Senampeline A	62787-00-2	C10388	C25H31NO8	473.204969	Alkaloids	9.23	+
476	Ruscogenin	472-11-7	C08909	C27H42O4	430.30831	Steroids and steroid derivatives	12.8	+
477	Syringic acid	530-57-4	C10833	C9H10O5	198.052825	Phenols	3.57	+
478	Guaiazulene	489-84-9	C09675	C15H18	198.14085	Sesquiterpenoids	8.74	+
479	Ethyl gallate	831-61-8		C9H10O5	198.052825	Phenols	8.86	+
480	Leukotriene A4	72059-45-1	C00909	C20H30O3	318.219495	Fatty Acyls	5.57	+
481	Wogonin	632-85-9	C10197	C16H12O5	284.068475	Flavonoids	11.08	+
482	6,7-Dehydroferruginol	34539-84-9		C20H28O	284.214015	Diterpenoids	9.06	+
483	Physcion	521-61-9	C17045	C16H12O5	284.068475	Anthraquinones	8.24	+
484	Genkwanin	437-64-9	C10046	C16H12O5	284.068475	Flavonoids	11.1	+
485	Prunetin	552-59-0	C10521	C16H12O5	284.068475	Flavonoids	11.07	+
486	Boldione	897-06-3	C20144	C19H24O2	284.17763	Steroids and steroid derivatives	9.07	+
487	Retinoic acid	302-79-4	C00777	C20H28O2	300.20893	Diterpenoids	8.46	+
488	Vitamin K1	84-80-0	C02059	C31H46O2	450.34978	Quinones	13.44	+
489	Phenylacetyl-L-glutamine	28047-15-6	C04148	C13H16N2O4	264.111008	Amino acid and derivatives	4.71	+
490	L-Norleucine	327-57-1	C01933	C6H13NO2	131.094629	Amino acid and derivatives	1.81	+
491	Isoleucine	443-79-8	C16434;C06418	C6H13NO2	131.094629	Amino acid and derivatives	1.8	+
492	5-Aminolevulinate	106-60-5	C00430	C5H9NO3	131.058244	Organic acids	1.56	+
493	Riboflavine	83-88-5	C00255	C17H20N4O6	376.138286	Vitamins	5.06	+
494	Lactacystin	133343-34-7		C15H24N2O7S	376.130424	Miscellaneous	9	+
495	Picrasin B	26121-56-2	C17050	C21H28O6	376.18859	Diterpenoids	4.46	+
496	5,7-Dihydroxyisoflavone	61-68-7	C02168	C15H15NO2	241.110279	Flavonoids	14.24	+
497	Eupatolide	6750-25-0	C09440	C15H20O3	248.141245	Sesquiterpenoids	7.18	+
498	1beta-Hydroxyalantolactone	68776-47-6		C15H20O3	248.141245	Sesquiterpenoids	5.26	+
499	Pterosin D	34169-70-5		C15H20O3	248.141245	Sesquiterpenoids	3.29	+
500	2-Picolinic acid	14639-25-9;98-98-6	C10164	C6H5NO2	123.032029	Organic acids	1.33	+
501	Isonicotinic acid	55-22-1	C07446	C6H5NO2	123.032029	Pyridines and derivatives	2.3	+
502	4-Hydroxybenzylamine	696-60-6		C7H9NO	123.068414	Alkaloids	2.3	+
503	Estriol	50-27-1	C05141	C18H24O3	288.172545	Steroids	5.78	+
504	Eriodictyol	552-58-9	C05631	C15H12O6	288.06339	Flavonoids	8.05	+
505	Shikonin	517-88-4	C10292	C16H16O5	288.099775	Quinones	5.14	+
506	Dalbergioidin	30368-42-4	C10415	C15H12O6	288.06339	Flavonoids	5.99	+
507	Micromelin	15085-71-9	C09277	C15H12O6	288.06339	Coumarins	5.13	+
508	Bovinic acid	2540-56-9	C04056	C18H32O2	280.24023	Fatty Acyls	6.86	+
509	Homoeriodictyol	446-71-9	C09756	C16H14O6	302.07904	Flavonoids	6.83	+
510	Kaurenoic acid	6730-83-2	C11874	C20H30O2	302.22458	Diterpenoids	5.9	+
511	Homoferreirin	482-01-9	C10457	C17H16O6	316.09469	Flavonoids	8.09	+
512	Darlingine	58471-10-6	C10857	C13H17NO2	219.125929	Alkaloids	11.13	+
513	Synephrine	94-07-5	C04548	C9H13NO2	167.094629	Alkaloids	2.63	+

514	Simvastatin	79902-63-9	C07262	C25H38O5	418.271925	Diterpenoids	8.07	+
515	Folic acid	59-30-3	C00504	C19H19N7O6	441.139683	Alkaloids	3.96	+
516	3-Isopropylmalate	921-28-8	C04411	C7H12O5	176.068475	Fatty Acyls	10.79	+
517	Ethyl cinnamate	103-36-6	C06359	C11H12O2	176.08373	Phenylpropanoids	4.85	+
518	Cinnamyl acetat	103-54-8	C12299	C11H12O2	176.08373	Phenols	10.56	+
519	Streptomycin	57-92-1	C00413	C21H39N7O12	581.265673	Organooxygen compounds	12.84	+
520	Dimethylbenzimidazole	582-60-5	C03114	C9H10N2	146.084398	Benzimidazoles	10.56	+
521	Ephedrine	299-42-3	C01575	C10H15NO	165.115364	Benzene and substituted derivatives	5.07	+
522	Benzocaine	94-09-7	C07527	C9H11NO2	165.078979	Benzene and substituted derivatives	3.05	+
523	7-Methylguanine	578-76-7	C02242	C6H7N5O	165.06506	Imidazopyrimidines	1.56	+
524	(R)-2-Hydroxy-2H-1,4-benzoxazin-3(4H)-one		C15769	C8H7NO3	165.042594	Benzoxazines	5.05	+
525	5-(3-Pyridyl)-2-hydroxytetrahydrofuran	53798-73-5	C19578	C9H11NO2	165.078979	Pyridines and derivatives	2.8	+
526	Cathinone	71031-15-7	C08301	C9H11NO	149.084064	Alkaloids	6.68	+
527	Epicatechin;(+)-Epicatechin	490-46-0;35323-91-2	C09727;C09728	C15H14O6	290.07904	Flavonoids	5.08	+
528	3-Ethoxy-4-hydroxybenzaldehyde;Ethyl salicylate	121-32-4;118-61-6		C9H10O3	166.062995	Phenols	5.72	+
529	Rhynchophylline;Isorhyncophylline	76-66-4;6859-01-4	C09236;C16980	C22H28N2O4	384.204908	Alkaloids	8.66	+
530	Euparin;Nepodin	532-48-9;3785-24-8	C09954	C13H12O3	216.078645	Phenols	8.74	+
531	Tropine acetate;3-Acetoxytropane	3423-27-6;3423-26-5	C12452;C12453	C10H17NO2	183.125929	Alkaloids	3.24	+
532	Citrostadienol	474-40-8	C11523	C30H50O	426.386165	Steroids	12.29	+
533	Reticuline;(R)-Reticuline	485-19-8;3968-19-2	C02105;C12328;C05178	C19H23NO4	329.162709	Alkaloids	5.24	+
534	Alizarin 2-methyl ether;Palmitoleic acid	6003-11-8;373-49-9	C10291;C08362	C15H10O4;C16H30O2	254.05791	Anthraquinones;Fatty Acyls	7.38	+
535	Vincamine;Luteone	1617-90-9;41743-56-0	C09251;C10498	C21H26N2O3;C20H18O6	354.194343	Alkaloids;Flavonoids	10.97	+
536	Kaempferol 3-O-beta-sophoroside;Cyanin	19895-95-5;2611-67-8;20905-74-2	C12634;C08639	C27H30O16	610.15339	Flavonoids	6.83	+
537	Isopulegol	7786-67-6;89-79-2		C10H18O	154.135765	Monoterpenoids	2.46	+
538	Cedrelone;Isomangiferin	1254-85-9;24699-16-9	C16979	C26H30O5;C19H18O11	422.209325	Triterpenoids;Xanthones	11.18	+
539	Beta-D-Glucose;alpha-D-Glucose	492-61-5;492-62-6	C00221;C00267	C6H12O6	180.06339	Organooxygen compounds	3.27	+
540	Ortho-Hydroxyphenylacetic acid;1,2-Epoxy-p-menth-8-ene	614-75-5;1195-92-2	C05852;C07271	C8H8O3;C10H16O	152.047345	Benzene and substituted derivatives;Oxepanes	5.91	+
541	L-Pipecolic acid;Pipecolic acid;(2E)-Decenoyl-ACP	3105-95-1;52-52-8	C00408;C03969	C6H11NO2	129.078979	Amino acid and derivatives;Carboxylic acids and derivatives	1.47	+
542	Isoquinoline;Quinoline	119-65-3;91-22-5	C06323;C06413	C9H7N	129.057849	Alkaloids;Quinolines and derivatives	6.13	+
543	Delphinidin-3-O-glucoside;Isoquercitrin	6906-38-3;50986-17-9;482-35-9	C12138	C21H20O12	464.09548	Flavonoids	6.06	+
544	25-Hydroxyvitamin D2-25-glucuronide;25-Hydroxyvitamin D2 25-(beta-glucuronide)		C03033	C34H52O8	588.36622	Organooxygen compounds	13.7	+
545	Amabiline;Supinine	17958-43-9;551-58-6	C10263;C10403	C15H25NO4	283.178359	Alkaloids	12.99	+
546	Gentisein;1,3,5-Trihydroxyxanthone	529-49-7;6732-85-0	C10065;C10094	C13H8O5	244.037175	Xanthones	8.73	+
547	Symlandine;Symphytine	74410-74-5;22571-95-5	C10408;C10409	C20H31NO6	381.215139	Alkaloids	13.01	+
548	Grandifloric acid;Steviol	22338-69-8;471-80-7	C17956;C20212	C20H30O3	318.219495	Diterpenoids	6.48	+
549	Retinal;9-cis-Retinal	116-31-4;514-85-2	C00376;C16681	C20H28O	284.214015	Prenol lipids	9.07	+
550	L-Isoleucine;L-Leucine	73-32-5;61-90-5	C00407;C00123	C6H13NO2	131.094629	Amino acid and derivatives	1.78	+
551	1,5-Anhydro-D-glucitol;trans-3-Hydroxycinnamic acid;p-Hydroxy-cinnamic acid	154-58-5;588-30-7;14755-02-3;501-98-4;7400-08-0	C16538;C07326;C12621;C00811	C6H12O5;C9H8O3	164.068475	Alcohols and polyols;Phenylpropanoids	5.07	+
552	Neogrifolin;Grifolin;Ugaxanthone	23665-96-5;6903-07-7;13179-11-8		C22H32O2;C18H16O6	328.24023	Phenols;Xanthones	13.8	+
553	Digiferruginol;Rubiadin;Anhydroglycinol	24094-45-9;117-02-2;67685-22-7	C10327;C10402;C10200	C15H10O4	254.05791	Anthraquinones;Phenols	10.59	+
554	alpha-Cyperol;Alismol;Spathulenol	20084-99-5;87827-55-2;6750-60-3	C16945;C17462	C15H24O	220.182715	Prenol lipids;Sesquiterpenoids	10.89	+
555	Physcion 1-O-beta-D-glucoside;Physcion 8-O-beta-D-monoglucoside;Sissotrin	26296-54-8;23451-01-6;5928-26-7	C10384;C05376	C22H22O10	446.1213	Anthraquinones;Flavonoids	6.7	+
556	Echinocystic acid;Pomolic acid;Sebiferenic acid	510-30-5;13849-91-7;94390-09-7	C08942	C30H48O4	472.35526	Triterpenoids	12.27	+
557	Oleic acid;Vaccenic acid;Petroselinic acid	112-80-1;693-72-1;593-39-5	C00712;C08367;C08363	C18H34O2	282.25588	Fatty Acyls	13.03	+
558	Geraniol;cis-3,7-Dimethyl-2,6-octadien-1-ol;(+)-alpha-Pinene;(-)-Linalool	106-24-1;106-25-2;7785-70-8;126-91-0	C01500;C09871;C06306;C11388	C10H18O;C10H16	154.135765	Monoterpenoids	10.54	+
559	Betulinic acid;Beta-boswellic acid;a,b-Elemolic acid;3-Epikatonic acid;Isomangiferolic acid;<br>Trametenolic acid;Bryonolic acid;3-Epioleanolic acid	472-15-1;631-69-6;28282-27-1;76035-62-6;13878-92-7;<br>24160-36-9;24480-45-3;25499-90-5	C08619;C08623	C30H48O3	456.360345	Triterpenoids	12.93	+
560	(S)-(-)-Limonene;(R)-(+)-Limonene;Alpha-pinene;Beta-pinene;alpha-Terpinene;<br>p-Mentha-1,4-diene;Gamma-terpinene;(+)-3-Carene;Borneol;Camphene;beta-Phellandrene	5989-54-8;5989-27-5;80-56-8;2437-95-8;18172-67-3;<br>99-86-5;99-85-4;498-15-7;507-70-0;79-92-5;555-10-2	C00521;C06099;C09880;C06307;C09898;<br>C09900;C11382;C01411;C06076;C19818	C10H16;C10H18O	136.1252	Monoterpenoids;Prenol lipids	10.56	+
561	3-Hydroxy-3-methylpentane-1,5-dioic acid	503-49-1	C03761	C6H10O5	162.052825	Amino acid and derivatives	2.32	-
562	Phlorizin	60-81-1	C01604	C21H24O10	436.13695	Chalcones	6.41	-
563	20-OH-Leukotriene B4	79516-82-8	C04853	C20H32O5	352.224975	Fatty Acyls	6.04	-
564	Maleic acid	110-16-7	C01384	C4H4O4	116.01096	Carboxylic acids and derivatives	0.93	-
565	Nodakenetin;Marmesin	13849-08-6;495-32-9	C09278;C09276	C14H14O4	246.08921	Coumarins	6.05	-
566	5'-Deoxy-5-fluorouridine	3094-09-5	C12739	C9H11FN2O5	246.065201	5'-deoxyribonucleosides	1.88	-
567	6-Shogaol	555-66-8	C10494	C17H24O3	276.172545	Phenols	4.8	-
568	Aflatoxin B1	1162-65-8	C06800	C17H12O6	312.06339	Coumarins and derivatives	7.77	-
569	Emodin	518-82-1	C10343	C15H10O5	270.052825	Anthraquinones	7.28	-
570	L-2-Hydroxyglutaric acid	13095-48-2	C03196	C5H8O5	148.037175	Fatty Acyls	4.12	-
571	Biochanin A	491-80-5	C00814	C16H12O5	284.068475	Flavonoids	11.38	-
572	Glycitein	40957-83-3	C14536	C16H12O5	284.068475	Flavonoids	11.06	-
573	Madecassic acid	18449-41-7		C30H48O6	504.34509	Triterpenoids	6.96	-
574	Ginkgolic acid C15:1	22910-60-7	C10794	C22H34O3	346.250795	Phenols	7.58	-
575	Grayanotoxin I	4720-09-6	C09103	C22H36O7	412.246105	Diterpenoids	8.63	-
576	Hematoxylin	517-28-2	C09931	C16H14O6	302.07904	Flavonoids	2.76	-
577	Carminic acid	1260-17-9	C11254	C22H20O14	508.08531	Anthraquinones	6.07	-
578	Hippuric acid	495-69-2	C01586	C9H9NO3	179.058244	Alkaloids	4.72	-
579	Loganic acid;Loganic acid	22255-40-9	C01512	C16H24O10	376.13695	Iridoids	7.86	-
580	Telmisartan	144701-48-4	C07710	C33H30N4O2	514.236876	Benzene and substituted derivatives	6.89	-
581	Alpha-Tocotrienol	58864-81-6;1721-51-3	C14153	C29H44O2	424.33413	Phenols	7.5	-
582	Cosmosiin;Apigenin 7-O-glucoside (Cosmosiin)	578-74-5	C04608	C21H20O10	432.10565	Flavonoids	6.07	-
583	1-Methyluric acid	708-79-2	C16359	C6H6N4O3	182.043991	Imidazopyrimidines	4.16	-
584	Hydroxyphenyllactic acid	306-23-0	C03672	C9H10O4	182.05791	Phenylpropanoids	5.04	-
585	D-Fructose 6-phosphate	643-13-0	C00085	C6H13O9P	260.029722	Carbohydrates	3.23	-
586	Hyperforin	11079-53-1	C07608	C35H52O4	536.38656	Miscellaneous	8.92	-
587	Malvidin 3-O-glucoside (Oenin);Malvidin 3-glucoside	18470-06-9	C12140	C23H24O12	492.12678	Flavonoids	7.3	-
588	4-Methylcatechol	452-86-8	C06730	C7H8O2	124.05243	Phenols	4.48	-
589	Helenalin	6754-13-8	C09473	C15H18O4	262.12051	Sesquiterpenoids	4.73	-
590	Cynarin;1,5-Dicaffeoylquinic acid	30964-13-7	C10445	C25H24O12	516.12678	Organic acids	6.72	-
591	Amarogentin	21018-84-8	C09767	C29H30O13	586.168645	Iridoids	7.96	-
592	Guaijaverin	22255-13-6		C20H18O11	434.084915	Flavonoids	7.75	-
593	Uridine 5'-diphosphate	58-98-0	C00015	C9H14N2O12P2	404.002202	Pyrimidine nucleotides	4.94	-
594	4-Pyridoxic acid	82-82-6	C00847	C8H9NO4	183.053159	Pyridine derivatives	2.5	-
595	Indolepyruvate	392-12-1	C00331	C11H9NO3	203.058244	Indoles and derivatives	3.03	-
596	Rhein	478-43-3	C10401	C15H8O6	284.03209	Anthraquinones	8.03	-
597	Hydrocortisone;Cortisol	50-23-7	C00735	C21H30O5	362.209325	Steroids and steroid derivatives	5.8	-

598	2-Naphthol	135-19-3	C11713	C10H8O	144.057515	Naphthalenes	10.23	-
599	Xanthohumol	6754-58-1	C16417	C21H22O5	354.146725	Flavanone	10.96	-
600	Taurine;2-Aminoethanesulfonic acid	107-35-7	C00245	C2H7NO3S	125.014665	Alkaloids	3.26	-
601	Gentisic acid	490-79-9	C00628	C7H6O4	154.02661	Phenols	3.62	-
602	Eugenol	97-53-0	C10453	C10H12O2	164.08373	Phenylpropanoids	4.77	-
603	Methylmalonic acid	516-05-2	C02170	C4H6O4	118.02661	Organic acids	3.23	-
604	Homogentisic acid	451-13-8	C00544	C8H8O4	168.04226	Phenols	4.69	-
605	Orsellinic acid	480-64-8	C01839	C8H8O4	168.04226	Phenols	4.23	-
606	3,4-Dihydroxyphenylacetic acid	102-32-9	C01161	C8H8O4	168.04226	Phenols	3.05	-
607	2'-Deoxyinosine;Deoxyinosine	890-38-0	C05512	C10H12N4O4	252.085856	Nucleotide and its derivates	4.34	-
608	2-Isopropylmalate	3237-44-3	C02504	C7H12O5	176.068475	Fatty Acyls	4.23	-
609	Deoxyguanosine	961-07-9	C00330	C10H13N5O4	267.096755	Nucleotide and its derivates	2.72	-
610	17a-Ethynylestradiol	57-63-6	C07534	C20H24O2	296.17763	Steroids and steroid derivatives	5.84	-
611	Phloretin; Phloretin	60-82-2	C00774	C15H14O5	274.084125	Chalcones	9.02	-
612	Catechin	154-23-4	C06562	C15H14O6	290.07904	Flavonoids	6.05	-
613	Tricetin	1621-84-7;520-31-0	C10192;C10079	C15H10O7	302.042655	Flavonoids	8.05	-
614	Taxifolin;Dihydroquercetin (Taxifolin)	480-18-2	C01617	C15H12O7	304.058305	Flavonoids	6.34	-
615	6beta-hydroxytestosterone;6beta-Hydroxytestosterone	62-99-7	C14497	C19H28O3	304.203845	Steroids and steroid derivatives	8.51	-
616	Xanthoxylin	90-24-4	C10726	C10H12O4	196.07356	Phenols	7.22	-
617	Prostaglandin E3	802-31-3	C06439	C20H30O5	350.209325	Fatty Acyls	4.81	-
618	Isoscopoletin;Scopoletin	776-86-3;92-61-5	C18079;C01752	C10H8O4	192.04226	Coumarins;Phenylpropanoids	6.21	-
